# High-Entropy Metal Diborides: A New Class of High-Entropy Materials and a New Type of Ultrahigh Temperature Ceramics

**DOI:** 10.1038/srep37946

**Published:** 2016-11-29

**Authors:** Joshua Gild, Yuanyao Zhang, Tyler Harrington, Sicong Jiang, Tao Hu, Matthew C. Quinn, William M. Mellor, Naixie Zhou, Kenneth Vecchio, Jian Luo

**Affiliations:** 1 Program of Materials Science and Engineering, University of California, San Diego, La Jolla, CA 92093-0448, USA; 2 Department of NanoEngineering, University of California, San Diego, La Jolla, CA 92093-0448, USA

## Abstract

Seven equimolar, five-component, metal diborides were fabricated via high-energy ball milling and spark plasma sintering. Six of them, including (Hf_0.2_Zr_0.2_Ta_0.2_Nb_0.2_Ti_0.2_)B_2_, (Hf_0.2_Zr_0.2_Ta_0.2_Mo_0.2_Ti_0.2_)B_2_, (Hf_0.2_Zr_0.2_Mo_0.2_Nb_0.2_Ti_0.2_)B_2_, (Hf_0.2_Mo_0.2_Ta_0.2_Nb_0.2_Ti_0.2_)B_2_, (Mo_0.2_Zr_0.2_Ta_0.2_Nb_0.2_Ti_0.2_)B_2_, and (Hf_0.2_Zr_0.2_Ta_0.2_Cr_0.2_Ti_0.2_)B_2_, possess virtually one solid-solution boride phase of the hexagonal AlB_2_ structure. Revised Hume-Rothery size-difference factors are used to rationalize the formation of high-entropy solid solutions in these metal diborides. Greater than 92% of the theoretical densities have been generally achieved with largely uniform compositions from nanoscale to microscale. Aberration-corrected scanning transmission electron microscopy (AC STEM), with high-angle annular dark-field and annular bright-field (HAADF and ABF) imaging and nanoscale compositional mapping, has been conducted to confirm the formation of 2-D high-entropy metal layers, separated by rigid 2-D boron nets, without any detectable layered segregation along the *c*-axis. These materials represent a new type of ultra-high temperature ceramics (UHTCs) as well as a new class of high-entropy materials, which not only exemplify the first high-entropy non-oxide ceramics (borides) fabricated but also possess a unique non-cubic (hexagonal) and layered (quasi-2D) high-entropy crystal structure that markedly differs from all those reported in prior studies. Initial property assessments show that both the hardness and the oxidation resistance of these high-entropy metal diborides are generally higher/better than the average performances of five individual metal diborides made by identical fabrication processing.

Recently, the fabrication and properties of metallic high entropy alloys (HEAs) have attracted significant research interests^1^,^2^. In an HEA, the configurational entropy of a solid-solution phase is maximized to stabilize it against the formation of intermetallics. Typically, five or more elements can be mixed in a HEA in equimolar concentrations to produce a maximum molar configurational entropy of Δ*S*_mix_ = *R*ln*N*, where *N* is the number of equimolar components and *R* is the gas constant^1^,^2^. HEAs have shown superior mechanical and physical properties^1^,^2^,^3^; specially, a series of recent studies fabricated a class of refractory, metallic HEAs and demonstrated their excellent wear resistance and strength, including (especially) exceptional high-temperature properties^4^,^5^,^6^,^7^,^8^. Since the minimization of Gibbs free energy (*G* = *H*−*TS*, where *H* is enthalpy, *S* is entropy, and *T* is temperature) dictates the thermodynamic stability of a material at a constant pressure, a high-entropy material (with large *S*) can be thermodynamically more stable (particularly) at high temperatures, motivating this study to explore the phase stability and fabrication feasibility of high-entropy metal diborides, as a new type of high-entropy materials as well as a new class of ultra-high temperature ceramics (UHTCs).

Most prior studies of crystalline high-entropy materials have been conducted for metallic HEAs of mostly simple face- and body-centered cubic (FCC and BCC), as well as occasionally hexagonal close packing (HCP), crystal structures^1^,^2^; much less studies have been done for making crystalline high-entropy ceramics (albeit that glasses can be considered high-entropy materials in a broad definition), particularly those with more complex, non-cubic, crystal structures. Most recently, Rost *et al*. successfully fabricated an entropy-stabilized oxide, (Mg_0.2_Co_0.2_Ni_0.2_Cu_0.2_Zn_0.2_)O, that possessed a single-phase rocksalt (which is also a FCC) structure when it was quenched from a sufficiently high temperature^9^; subsequent studies revealed that this entropy-stabilized oxide and its derivatives, (Mg_0.2_Co_0.2_Ni_0.2_Cu_0.2_Zn_0.2_)_1-*x*-*y*_Ga_*y*_*A*_*x*_O (where *A* = Li, Na, or K), have colossal dielectric constants^10^ and superionic conductivities^11^. To the best of our knowledge, this class of entropy-stabilized oxides and its derivatives represent the first and only crystalline high-entropy ceramics that have been reported to date.

This study further extended the state of the art for the crystalline high-entropy ceramics via successfully synthesizing a new class of high-entropy metal diborides, including (Hf_0.2_Zr_0.2_Ta_0.2_Nb_0.2_Ti_0.2_)B_2_, (Hf_0.2_Zr_0.2_Ta_0.2_Mo_0.2_Ti_0.2_)B_2_, (Hf_0.2_Zr_0.2_Mo_0.2_Nb_0.2_Ti_0.2_)B_2_, (Hf_0.2_Mo_0.2_Ta_0.2_Nb_0.2_Ti_0.2_)B_2_, (Mo_0.2_Zr_0.2_Ta_0.2_Nb_0.2_Ti_0.2_)B_2_, and (Hf_0.2_Zr_0.2_Ta_0.2_Cr_0.2_Ti_0.2_)B_2_ (Table 1). This work has greatly extended the knowledge of high-entropy materials, not only since it is the first time crystalline high-entropy non-oxide ceramics (specifically borides) have been synthesized, but also because these high-entropy metal diborides exhibit a unique layered hexagonal crystal structure with alternating rigid two-dimensional (2D) boron nets and *high-entropy 2D layers* of metal cations (as essentially a class of quasi-2D high-entropy materials), as schematically shown in Fig. 1, which distinctly differs from any other high-entropy crystalline phases reported to date.

## Results

### Phase Evolution and Formation of High-Entropy Ceramic Phases

To synthesize high-entropy metal diborides, five commercial metal diboride powders of equimolar amounts were mixed and mechanically alloyed via high energy ball milling (HEBM) for six hours; subsequently, the HEBM powders were compacted into disks of 20-mm diameter and densified utilizing spark plasmas sintering (SPS) at 2000 °C for 5 minutes under a pressure of 30 MPa. The detailed synthesis procedure was described in the “Methods” section. Seven high-entropy metal diboride compositions were tested in this study, which are sometimes referred as HEB #1-#7 (as listed in Table 1 and Supplementary Table S-I. Representative X-ray diffraction (XRD) patterns shown in Fig. 2 and Supplementary Figs S1–S7 illustrate the phase evolution during the HEBM and SPS fabrication process. The initial mixture of powder displayed XRD peaks for five individual metal diboride phases (although some peaks overlap for most compositions), which broadened and merged (due to the particle size reduction and mechanical alloying effects during HEBM); eventually, a single, high-entropy, phase of the hexagonal AlB_2_ structure formed after SPS at 2000 °C (Fig. 2; Supplementary Figs S1–S7). Full-range XRD patterns of the SPS specimens are displayed in Fig. 3 (and in expanded views in Supplementary Figs S1–S7), where six of them, *i.e.*, (Hf_0.2_Zr_0.2_Ta_0.2_Nb_0.2_Ti_0.2_)B_2_, (Hf_0.2_Zr_0.2_Ta_0.2_Mo_0.2_Ti_0.2_)B_2_, (Hf_0.2_Zr_0.2_Mo_0.2_Nb_0.2_Ti_0.2_)B_2_, (Hf_0.2_Mo_0.2_Ta_0.2_Nb_0.2_Ti_0.2_)B_2_, (Mo_0.2_Zr_0.2_Ta_0.2_Nb_0.2_Ti_0.2_)B_2_, and (Hf_0.2_Zr_0.2_Ta_0.2_Cr_0.2_Ti_0.2_)B_2_, exhibit largely a single hexagonal phase, albeit the presence of minor secondary (Zr, Hf)O_2_ phases; these secondary oxide phases are represented by the low-intensity peaks that are evident in Figs 2 and 3, which are not indexed in Figs 2 and 3 for the figure clarity, but indicated by the solid dots in Supplementary Figs S1–S7. The formation of minor amounts of secondary oxide (ZrO_2_ or HfO_2_) phases is commonly observed in sintered ZrB_2_ and HfB_2_ specimens, which are native oxides that are difficult to remove (because of the extreme stabilities of native oxides of ZrO_2_ and HfO_2_). As the only special case, a secondary boride phase was observed in HEB #6, (Hf_0.2_Zr_0.2_W_0.2_Mo_0.2_Ti_0.2_)B_2_, with XRD peaks matching those of the (Ti_1.6_W_2.4_)B_4_ compound, while the major XRD peaks still represent a hexagonal metal diboride solid-solution phase (Fig. 3 and Supplementary Fig. S6).

### Compositional Uniformity

Cross-sectional scanning electron microscopy (SEM) images and the corresponding energy dispersive X-ray (EDX) spectroscopy compositional maps of three selected specimens (after SPS at 2000 °C) are shown in Fig. 4 (additional EDX compositional maps of all seven specimens are documented in the Supplementary Figs S1–S7). The compositions of all specimens are largely uniform, albeit the presence of uniformly-distributed minor secondary (Zr, Hf)O_2_ phases (to different extents in different specimens), as well as the (Ti_1.6_W_2.4_)B_4_ secondary phase in HEB #6 (only). Less than 1 at.% W (tungsten) is present in Specimens #1-#5 and #7 as contamination from the WC-based milling media used in HEBM. EDX mapping operating at 20 kV also found micrometer-scale Nb (niobium) localization in Specimens #1 and #3-#5, with occasional Zr and Mo clustering occurring concurrently in the same regions. This is somewhat surprising considering the fact that NbB_2_ generally forms continuous solid solutions with other metal diborides^12^. Presumably, the Nb localization is due to kinetic effects and can be homogenized with annealing for a prolonged time or at higher temperatures. In general, the compositional homogeneities are largely satisfactory, as shown in Fig. 4 (and in expanded views in Supplementary Figs S1–S7 for all seven specimens); they are significantly more homogenous than the typical (BCC) refractory HEAs made by casting, which usually form dendrite structures with severe compositional segregations^7^.

### Atomic-Resolution Structural Characterization

**Figure 1:**
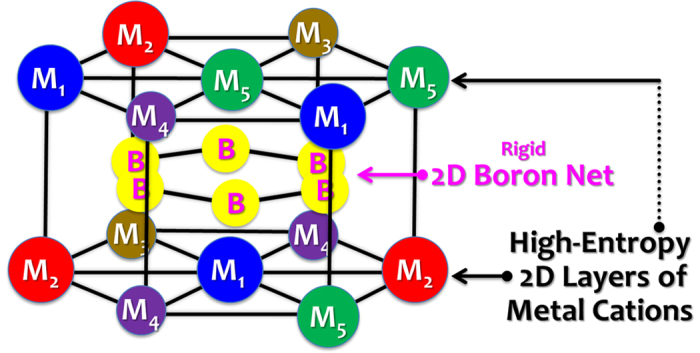
Schematic illustration of the atomic structure of the high-entropy metal diborides. Here, M_1_, M_2_, M_3_, M_4_, and M_5_ represent five different transition metals (selected from Zr, Hf, Ti, Ta, Nb, W, and Mo). This new class of high-entropy materials and new type of UHTCs have a unique layered hexagonal crystal structure with alternating rigid 2D boron nets and high-entropy 2D layers of metal cations (as essentially a class of quasi-2D high-entropy materials), with mixed ionic and covalent (M-B) bonds between the metals and boron.

AC STEM HAADF and ABF imaging has been conducted to confirm the formation of uniform solid solution at nanometer and atomic scales, particularly the formation 2-D high-entropy metal layers (separated by the rigid 2-D boron nets in the (0001) basal planes) without any significant layer-to-layer variation (or layered segregation) of different metal atoms in different (0001) planes perpendicular to the *c*-axis. The STEM-ABF and STEM-HAADF images in Fig. 5(a) and (b) show a homogeneous solid solution phase in the HEB #2, (Hf_0.2_Zr_0.2_Ti_0.2_Mo_0.2_Ta_0.2_)B_2_. STEM ABF and HAADF images with higher magnification showed the atomic configuration of atoms in the view of 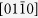 zone axis. The atomic planes (0001) and 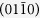 were indicated in Fig. 5(c). The mean spacing between two (0001) planes is about 3.449 Å, which is close to 3.316 Å measured by XRD. In Fig. 5(c), the metallic atoms were highlighted by red circles on (0001) plane. Light element B can be visualized via ABF imaging. The highlighted green dots in Fig. 5(c) indicated the B atoms, which are located between two basal planes (0001). The observed atomic configuration is consistent with the unit cell model depicted in Fig. 1. The same atomic configuration and homogeneity were also observed in different locations (Figs S8 and S9) and a different specimen (Fig. S10). A careful digital image analysis (Fig. S11) revealed that the measured standard deviations of lattice spacings between the basal (0001) planes are only ~0.6% of the average measured *c* lattice parameter or the measured variations from STEM ABF and HAADF images are ~0.02 Å, which directly confirmed the formation 2-D high-entropy metal layers without a layered segregation of different metal specimens in different (0001) basal planes, where these 2-D metal layers are well separated by the rigid 2-D boron nets in between (Fig. 1). Thus, these high-entropy metal diborides can be considered as (layered) quasi-2D high-entropy materials, as schematically illustrated in Fig. 1.

### Nanoscale Compositional Mapping

The compositional homogeneity at nanoscale for the HEB #2, (Hf_0.2_Zr_0.2_Ti_0.2_Mo_0.2_Ta_0.2_)B_2_, was confirmed by EDX maps for different metallic elements. Figure 6 showed that Hf, Zr, Ta, Mo and Ti were uniformly distributed at nanoscale. No segregation or aggregation was found throughout the scanned area. Since these compositional maps were also taken with the electron beam being parallel to the 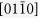 zone axis, they also confirmed no layered segregation along the *c*-axis in (0001) basal planes; thus, this is indeed a quasi-2D high-entropy material as illustrated in Fig. 1. Additional EDX mapping at a different location was also conducted and documented in Fig. S11.

### Densification and Lattice Parameters

**Figure 2:**
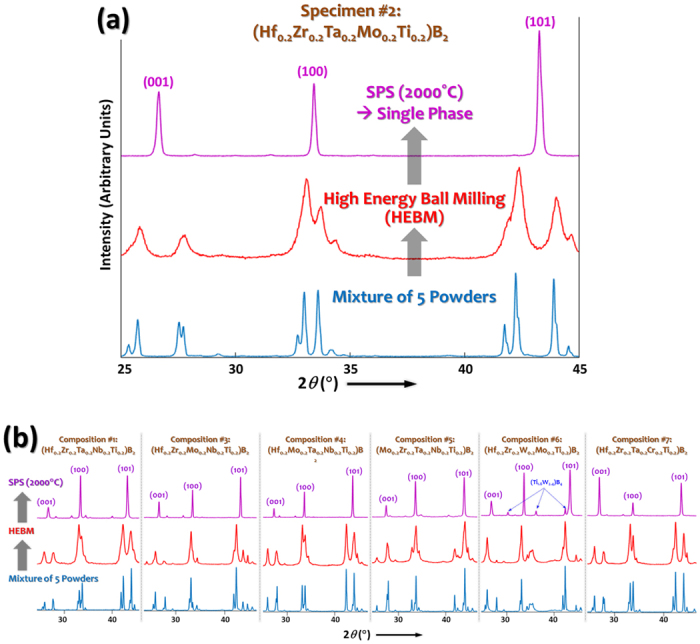
XRD patterns showing the phase evolution during the HEBM and SPS fabrication processes in (**a**) HEB #2 as an examplar in an expanded scale and (**b**) six other specimens. Only the first three peaks of the high-entropy hexagonal AlB_2_ phases are shown here for figure clarity; full-range XRD patterns (of 2θ = 20°–100°, showing eleven XRD peaks of the high-entropy hexagonal phases) are documented in the Supplementary Figs S1–S7.

**Figure 3:**
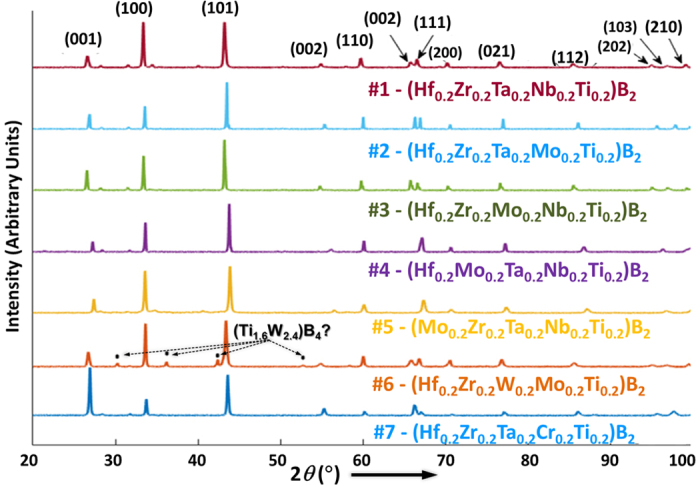
XRD patterns of all seven specimens after SPS at 2000 °C, where the peaks of the primary hexagonal phase are indexed. Six of seven specimens (except for HEB #6) exhibit largely a single hexagonal phase of the AlB_2_ structure, albeit the presence of minor secondary (Zr, Hf)O_2_ (native oxides), which are represented by the low-intensity peaks that are not indexed here the figure clarity (but indicated by the solid dots in Supplementary Figs S1–S7). As the only special case, a secondary boride phase was observed in HEB #6, with XRD peaks matching those of the (Ti_1.6_W_2.4_)B_4_ compound, while the major XRD peaks still represent a hexagonal metal diboride solid-solution phase.

**Figure 4:**
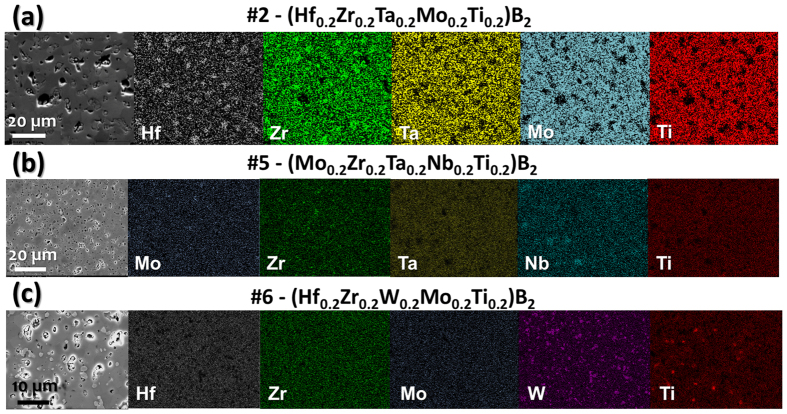
Cross-sectional SEM image and the corresponding EDX compositional maps of three selected specimens after SPS, showing the formation of largely homogeneous high-entropy solid-solution phases except for the HEB #6 shown in (**c**). The compositions are largely uniform albeit the presence of minor (Zr, Hf)O_2_ based native oxides, e.g., in (**a**), and some Nb clustering in four Nb-containing specimens, e.g., in (**b**). The formation of a secondary boride phase was observed only in HEB # 6, as shown in (**c**). Additional EDX compositional maps (in expanded views) of all seven specimens are documented in the Supplementary Figs S1–S7.

**Figure 5:**
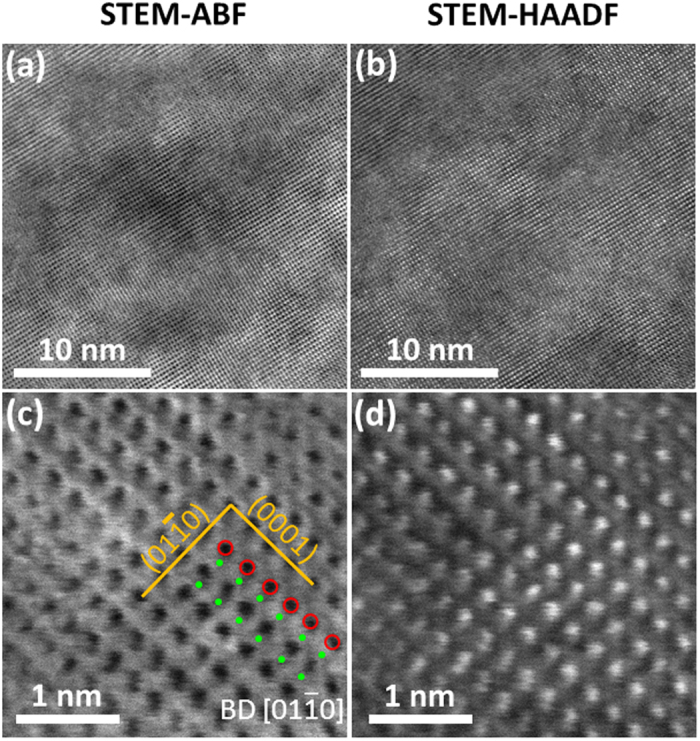
Atomic-resolution STEM ABF and HAADF images of HEB #2 (Hf_0.2_Zr_0.2_Ta_0.2_Mo_0.2_Ti_0.2_)B_2_. (**a**) and (**b**): ABF and HAADF images at a low magnification, showing the homogeneity of the solid-solution phase. (**c**) and (**d**): ABF and HAADF images at a higher magnification, showing atomic configuration. The electron beam is parallel to the 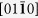 zone axis of hexagonal structure. (0001) and 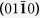 planes are indexed in (**c**). The red circles highlight the columns of transition metal atoms (Hf, Zr, Ta, Mo and Ti). The green dots indicate the B atoms. Additional STEM images from different regions and a different specimen are documented in the Supplementary Figs S8–S10; a further digital analysis of HAADF and ABF images in Supplementary Fig. S11 shows that the standard variations in the (0001) lattice spacings are only ~0.6% or ~0.02 Å, indicating homogenous mixing of five metal atoms (Hf, Zr, Ta, Mo and Ti) within the 2-D metal layers in (0001) planes.

**Figure 6:**
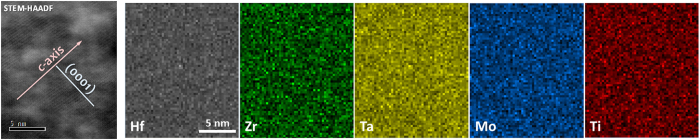
STEM-HAADF image and the corresponding EDS compositional maps for HEB #2 (Hf_0.2_Zr_0.2_Ta_0.2_Mo_0.2_Ti_0.2_)B_2_, showing the homogeneous chemical distribution at nanoscale. These compositional maps were taken when the electron beam is parallel to the 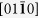 zone axis, showing no significant layer-to-layer variations of metal composition in different basal (0001) planes. Additional EDX compositional maps obtained from a different region are documented in the Supplementary Fig. S12.

In general, greater than 92% of theoretical densities has been achieved by SPS at 2000 °C (Table 1; see Supplementary Table S-I for the actual measured densities, along with the theoretical densities calculated using the lattice parameters measured by XRD). The lattice parameters were measured from XRD and listed in Table 1. Typically, the measured lattice parameters are within <1% of those calculated by the rule of mixtures (Table 1), which, along with the narrow XRD peaks (where the peak widths are much narrower than the mean differences among the five peaks of individual metal diborides, as shown in Fig. 2 and Supplementary Figs S1–S7), indicates the formation of disordered solid solutions for all high-entropy metal diborides made in this study (consistent with the direct STEM HAADF/ABF imaging and nanoscale compositional mapping as shown in Figs 5 and 6).

### Hardness and Oxidation Resistance

**Figure 7:**
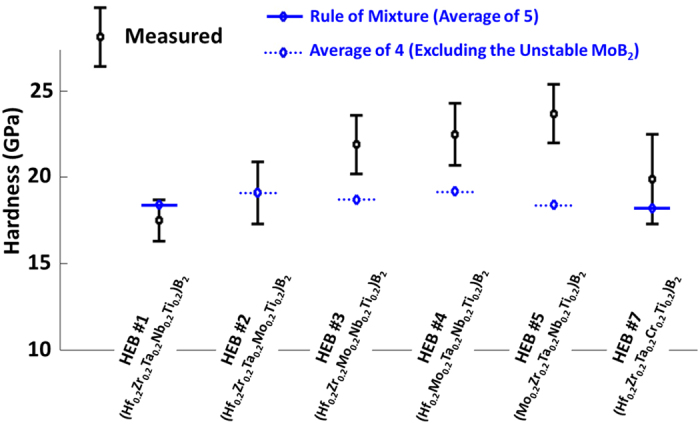
Measured hardness of six single-phase high-entropy metal diborides, which are generally greater than the “rule-of-mixtures” averages of the hardness values measured from individual metal diborides that were fabricated via the same HEBM and SPS route. Since MoB_2_ is not an equilibrium bulk phase below 1500 °C, the averages for HEB#2-HEB#5 were calculated without MoB_2_. However, MoB_2_ has a lower melting temperature and theoretical hardness than all other five other metal diborides in question; thus, the actual rule-of-mixtures averages should be even lower. It is also important to note that the hardness can be affected by porosity and oxide inclusions so that fully-dense and oxide-free metal diborides should have greater hardness than these measured values. We choose to compare the measured hardness values of high-entropy and conventional metal diborides fabricated by the same method to allow a fair assessment of relative values.

**Figure 8:**
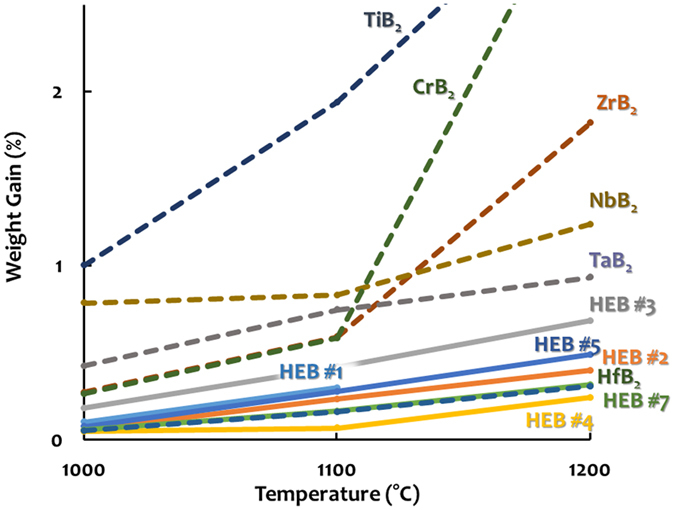
A snapshot of the relative oxidation performance of various high-entropy and individual metal diborides fabricated and tested with the same conditions. This figure displays percentage weight gain vs. oxidation temperature curves during annealing in flowing dry air at 1000 °C, 1100 °C, and 1200 °C (for one hour each) sequentially for six single-phase high-entropy metal diborides [HEB #1 = (Hf_0.2_Zr_0.2_Ta_0.2_Nb_0.2_Ti_0.2_)B_2_, HEB #2 = (Hf_0.2_Zr_0.2_Ta_0.2_Mo_0.2_Ti_0.2_)B_2_, HEB #3 = (Hf_0.2_Zr_0.2_Mo_0.2_Nb_0.2_Ti_0.2_)B_2_, HEB #4 = (Hf_0.2_Mo_0.2_Ta_0.2_Nb_0.2_Ti_0.2_)B_2_, HEB #5 = (Mo_0.2_Zr_0.2_Ta_0.2_Nb_0.2_Ti_0.2_)B_2_, and HEB #7 = (Hf_0.2_Zr_0.2_Ta_0.2_Cr_0.2_Ti_0.2_)B_2_], along with six individual metal diborides fabricated via the same HEBM and SPS route. See the “Methods” section for the experimental procedure and Supplementary Figs S13–S15 for additional results, including weight gain per surface area plots, weight percentage gains at higher temperatures, and images of all specimens after oxidation at different temperatures. In this figure (and Supplementary Fig. S13), solid lines represent the high-entropy metal diborides and dashed lines represent the individual (conventional) metal diborides made by the same fabrication route.

Initial property assessments indicated that both the hardness and the oxidation resistance of these high-entropy metal diborides are generally greater or better than the average performances of the individual (conventional) metal diborides made by the identical HEBM and SPS fabrication processing. We understand that both hardness and oxidation resistance should critically depend on microstructures; the presence of porosity and oxide inclusion, as a consequence of the HEBM procedure that we adopted for promoting the homogenization of high-entropy solid solutions, adversely affected the hardness and oxidation resistance. To conduct a fair assessment of the relative performance of high-entropy and conventional metal diborides, we measured six single-phase high-entropy diborides, along with a controlled group of HfB_2_, ZrB_2_, TaB_2_, NbB_2_, TiB_2_, and CrB_2_ specimens made by the identical HEBM and SPS fabrication processing using the same processing parameters (except for CrB_2_; see “Methods” section for explanation). Figure 7 displays the measured hardness of six high-entropy metal diborides (with the actual measured data being listed in Supplementary Table S-III), which are generally greater than the averages of the hardness values measured from individual metal diborides fabricated via the same route. Because MoB_2_ is not an equilibrium bulk phase below 1500 °C, the averages for HEB#2-HEB#5 that contains 20% MoB_2_ were calculated without MoB_2_. Yet, it is well established that MoB_2_ has a lower melting temperature and theoretical hardness than all the other metal diborides in HEB#2-HEB#5 (HfB_2_, ZrB_2_, TaB_2_, NbB_2_, and TiB_2_) so that the actual averages from the “rule of mixtures,” if we could make and measure MoB_2_ via the same procedure, should be even lower. Furthermore, results from an initial oxidation resistance measurement of these high-entropy and individual metal diborides made by the identical fabrication processing are shown in Fig. 8, with additional data and images documented in Supplementary S13–S15. Taking HEB#1 (Hf_0.2_Zr_0.2_Ta_0.2_Nb_0.2_Ti_0.2_)B_2_ as an example (which is a good case for considering because none of its oxides is volatile in this temperature range so that the weight gains shown in Fig. 8 and Fig. S13 are easier to interpret), Figs 8, S13 and S14 show that HEB#1 performs better than most of its individual components made with the same procedure (ZrB_2_, TaB_2_, NbB_2_, and TiB_2_) except for HfB_2_; it certainly performs better than the “average” performance of these five individual metal diborides. Consistently, both HEB #1 and HEB #7 maintained their shapes even at 1500 °C, while the majority of the respective individual metal diborides (except for HfB_2_) that were fabricated via the same HEBM and SPS route oxidized more severely. For example, the TiB_2_ specimen, which represents one most widely-used metal diboride today, pulverized completely at 1500 °C (Supplementary Fig. S14). Finally, the four MoB_2_-containing high-entropy diborides (HEB#2-HEB#5) exhibited interesting and diverse, oxidation behaviors because MoO_3_ is volatile. Despite this, some of them still perform better than many conventional metal diborides that do not have volatile native oxides (Figs S13 and S15).

## Discussion

The formation of (metallic) HEAs are often predicted by using the atomic-size effect (*δ*) and the enthalpy of mixing (Δ*H*_mix_) as the two main criteria^1^,^2^. The enthalpy of mixing is difficult to quantify for the current case, so attention is focused on analyzing the atomic-size effect. The original Hume-Rothery solid-solution rule suggests that (*r*_solute_−*r*_solvent_)/*r*_solvent_ ≤ 15% is one of the necessary conditions for forming a binary solid solution. Following the same concept, the average atomic-size difference (*δ*) can be defined for a multicomponent HEA alloy^1^,^2^, as:


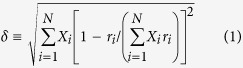


where *r*_i_ and *X*_i_ are the atomic radius and molar content, respectively, of the *i*-th component. Prior studies suggested, mostly based on empirical observations, that a necessary (but not sufficient) criterion for forming a single-phase (disordered) HEA is that the computed *δ* of the solid solution should be sufficiently small: *δ* ≤ *δ*_max_ ≈ 4%^1^ or 4.3%^2^. By simply plugging the values of metallic or covalent radii of the metals, and the computed *δ* values are in the range of 3.5% to ~8% (Table S-I in the Supplementary Material); specifically, HEB #7 has the highest *δ* ≈ 8%; yet, it still forms single-phase, high-entropy, solid solution. In reality, metal diborides [M^2+^(B^−^)_2_] form a highly anisotropic layered structure (*i.e.*, the hexagonal AlB_2_ structure^13^), where each metal atom donates two electrons and the M-B bonds (between the metal and B layers) have mixed ionic and covalent characteristics (see Fig. 1). Within the 2D metal layers, M-M bonds are strained significantly by the more rigid boron net (Fig. 1). Thus, none of the available (metallic, covalent or ionic) radii can effectively represent the actual bond lengths in the metal diborides in the AlB_2_ structure (Fig. 1)^13^.

Alternatively, we propose to calculate the average size difference for a high-entropy metal diboride using the lattice constants of individual metal diborides (measured lattice parameters *a*_i_ and *c*_i_ for the *i*-th MB_2_, as summarized in ref. 14, instead of the atomic radii of metals), as:


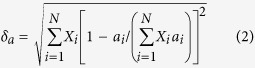


and


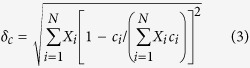


Subsequently, the values of *δ*_*a*_ and *δ*_*c*_ have been computed for the seven specimens and listed in Table 1 and Supplementary Table S-I. Interestingly, the computed *δ*_*a*_ values are small (in the range of 1.3% to 2.3% for all seven specimens) because the M-M bonds are strained by the rigid boron net (that can deform metal cations and M-M bond lengths towards an ideal “strain-free” value dictated by stronger B-B bonds^13^; Fig. 1). Thus, the computed *δ*_*c*_ values may better represent the average size difference because of less constraint along the *c*-axis. Coincidentally, Specimens #1-#5 and #7, for which single-phase, high-entropy, solid solutions did form, all have computed *δ*_*c*_ values in the range of 3.9% to 5.2%, whereas HEB #6, for which single-phase did not form, has the largest computed *δ*_*c*_ value of ~6.2%. It is interesting to further note that HEB #7 (with a simple high-entropy phase) has a greater *δ*_*a*_but smaller *δ*_*c*_than those of HEB #6 (with two boride phases), suggesting that a smaller *δ*_*c*_ may be more important than a smaller *δ*_*a*_.

However, we should emphasize that small differences in lattice parameters (measured by small *δ*_*a*_and *δ*_*c*_) are only one necessary, but not essential, condition for forming high-entropy solutions. A very small *δ* value is certainly not a guarantee for forming a single-phase, high-entropy, solid solution. For example, the precipitation of the secondary (Ti_1.6_W_2.4_)B_4_ phase in HEB #6 may be related to the facts that this (Ti_1.6_W_1.4_)B_4_ phase is extremely stable or WB_2_ is not stable by itself; further investigation is needed here to clarify the most important reason for the precipitation of (Ti_1.6_W_2.4_)B_4_ in HEB #6.

Moreover, the average size differences are certainly not the only factors that determine the ability to form a single high-entropy phase. For example, it is known^13^ that an average lattice parameter *a* of ~3.04 Å would produce “strain-free” metal layers that match the rigid boron net, thereby being favored; this may also be a factor for HEB #7 to exhibit single high-entropy phase since its average *a* (of ~3.081 Å) has the closest match to the ideal strain-free value (Supplementary Table S-I; despite that this factor also favors HEB #6, where the largest *δ*_*c*_may be a determining factor). In addition to the several size factors discussed above, the mixing enthalpy, as well as the valence electron concentration, may also play an important role in determining whether a single high-entropy phase forms^1^,^2^.

It is worth making a few additional notes regarding the observed phase stabilities. First, perhaps the most interesting observation is the formation of a single-phase, high-entropy solution in HEB #7, (Hf_0.2_Zr_0.2_Ti_0.2_Cr_0.2_Ta_0.2_)B_2_, despite the limited solid solubilities of CrB_2_ in both HfB_2_ and ZrB_2_^12^,^15^. Second, MoB_2_ is believed to be metastable at room temperature, but the hexagonal MoB_2_ phase could be retained in the SPS specimens^16^; in this study, four 20%-Mo-containing high-entropy metal diborides have been made. Third, the starting powder W_2_B_5_ (since WB_2_ is not commercially available) possessed a different structure and it has limited solubilities in all diborides except for TiB_2_^17^,^18^,^19^, which can be another reason that HEB #6 did not possess a single solid-solution phase (in addition to the largest *δ*_*c*_ of ~6.2%).

It is important to emphasize that both the hardness and oxidation resistance can be affected by the microstructure, *e.g.*, the porosity and oxide inclusions, significantly. Thus, we choose to compare the high-entropy and individual metal diborides fabricated using the same method to allow a fair assessment of relative hardness and oxidation resistance (even if our specimens have high levels of porosity and oxidation inclusions due to HEBM than those fully-dense and oxide-free specimens prepared by other fabrication routes). We expect that fully-dense and oxide-free specimens should have higher hardness and better oxidation resistance.

Although the high-entropy metal diborides do appear to exhibit greater hardness and better oxidization resistance than the average performances of the individual metal diborides (provided that they are made with the same fabrication route), perhaps a more important advantage for adopting high-entropy materials is a large compositional design space to allow tuning of properties. This will be particularly important for improving oxidation resistance, which depends on many (often kinetic) factors; thus, there is perhaps no simple answer on whether high-entropy metal diborides are good or bad for oxidation resistance (and some other properties). A large compositional design space is useful for designing better protective oxide scales (with additives or in composites, which are often necessary for real applications), representing a complex material engineering problem beyond the scope of this study. Further systematic investigation of hardness, oxidation resistance, and other properties of the high-entropy metal diborides, which often critically depend on the microstructure and therefore the processing optimization, is important but beyond the scope of this study that focuses on the formation, structure, microstructure, and thermodynamic stability of this new class of high-entropy materials.

In summary, this study has successfully synthesized six single-phase, high-entropy, metal diborides via mechanical alloying and SPS. In general, metal diboride-based UHTCs have ultrahigh melting points, as well as excellent thermal and electrical conductivities, hardness, and wear and oxidation resistances^13^,^15^,^20^,^21^,^22^,^23^; thus, they have potential structural applications in extreme environments. In addition, with a unique, layered hexagonal (AlB_2_) crystal structure, with alternating metal and boron layers, some metal diborides also exhibit exotic functionality, *e.g.*, MgB_2_ is a well-known superconductor. While extensive future research has to be conducted to investigate their mechanical, chemical (oxidation), and physical properties, these high-entropy metal diborides represent a new class of UHTCs, as well as a new type of high-entropy materials that can have unique compositions and structures that differ distinctly from any other existing materials, as well as great possibilities of tailoring their properties via an extremely-large compositional engineering space.

## Methods

### Synthesis of High-Entropy Metal Diboride Specimens

To synthesize high-entropy metal diborides, powders of HfB_2_, ZrB_2_, NbB_2_, TiB_2_, W_2_B_5_ (to substitute WB_2_ that is not commercially available), CrB_2_ (99.5% purity; purchased from Alfa Aesar, MA, USA), TaB_2_, and MoB_2_ (99% purity; purchased from Goodfellow, PA, USA) were utilized as starting materials. Appropriate amounts of five powders were utilized to fabricate specimens of each composition (with the stoichiometry being calculated on the metal basis). The seven compositions are listed in Table 1 and referred to as HEB #1 to #7 in the text. The raw powders were mechanically alloyed via high energy ball milling (HEBM) using a Spex 8000D mill (SpexCertPrep, NJ, USA) for six hours in WC media. To prevent overheating, the HEBM was stopped every 60 minutes to allow cooling for five minutes. The powders were then hand ground in an agate mortar to a 325 mesh; subsequently, they were compacted into disks of 20-mm diameter and densified utilizing spark plasma sintering (SPS, Thermal Technologies, CA, USA) in vacuum (10^−2^ Torr) at 2000 °C for 5 minutes under a pressure of 30 MPa, with a heating ramp rate of 100 °C/min. The inside of the graphite die was lined with a 25μm-thick molybdenum foil to prevent reactions between the graphite and the diboride specimen. The molybdenum foil was then lined with a layer of 125μm-thick graphite paper to minimize reactions between the foil and the outer die.

### Characterization

The specimens were characterized by X-ray diffraction (XRD) using a Rigaku diffractometer with Cu Kα radiation and scanning electron microscopy (SEM) in conjunction with energy dispersive X-ray spectroscopy (EDX). The specimen densities were measured via the Archimedes method to an accuracy of ±0.01 g/cm^2^ and the relative densities were calculated via using theoretical densities that were determined by the ideal stoichiometry and lattice parameters measured by XRD. The atomic and nanoscale characterization was conducted using aberration-corrected scanning transmission electron microscopy (AC STEM); STEM high-angle annular dark-field (HAADF) images, medium-angle annular dark-field (MAADF), and annular bright-field (ABF) images were taken by using a 200 kV STEM (ARM-200F, JEOL) equipped with a probe Cs corrector (CEOS Gmbh), which offers an unprecedented opportunity to probe structures with a sub-Ångström resolution. For HAADF imaging, we adopted a probe convergence angle of ~22 mrad and a detector with inner semi-angle of >60 mrad. The ABF images were taken with a detector of 12–23 mrad, while MAADF images were taken with a detector of 23–50 mrad. The energy dispersion X-ray (EDX) spectroscopy was employed to map the chemical composition at nanoscale. The TEM samples were prepared by dual-beam FIB/SEM system (Scios, FEI).

### Hardness and Oxidation Measurements

Hardness and oxidation measurements were conducted using all six single-phase high-entropy diborides (HEB #1-#5 and #7) and six individual metal diboride benchmarking specimens (HfB_2_, ZrB_2_, TaB_2_, NbB_2_, TiB_2_, and CrB_2_) that were made by the same HEBM and SPS fabrication method using the same processing parameters, with one exception that CrB_2_ was sintered at lower temperature of 1800 °C because its substantially lower melting (and therefore sintering) temperature. MoB_2_ was not examined because it is not a thermodynamically stable phase (and will decompose to MoB and Mo_2_B_5_) below 1500 °C. Hardness measurements were performed with a Vickers’ diamond indenter at 200 kgf/mm^2^ with a hold time of 15 seconds. The indentations were examined for conformation with the ASTM C1327. The indentations averaged 20–25 μm in width during the testing. Multiple measurements were performed at different locations of each specimen; the mean and standard deviation are reported. The density and hardness are generally uniform at different locations for HEB specimens #1-#5 and all six individual metal diboride specimens; however, HEB #7 has a denser outside shell and less dense inner core with different average hardness values (due to the effect of low-melting CrB_2_ that promotes rapid densification near the surface); thus, the hardness values were measured at both regions and reported in Supplementary Table S-III but only the overall mean and standard deviation were used in comparison. The oxidation experiments were conducted in a tube furnace under flowing dry air. The specimens were annealed at 800 °C, 900 °C, 1000 °C, 1100 °C, 1200 °C, 1300 °C, 1400 °C, and 1500 °C sequentially. Each annealing step included a one-hour isothermal oxidation at the desired temperature with a heating ramp rate of 10 °C/min; after the isothermal annealing, the specimens were cooled in the furnace with uncontrolled cooling rates on the order of 100 °C/min. After each annealing step, the specimens were removed from the furnace and weighted. At low annealing temperatures, specimens were weighted directly. At high temperatures (typically1300 °C and above), many specimens reacted with the alumina crucibles so that the specimens were weighted in the crucibles to obtain the net weight gains/losses. We found the measured weights are generally accurate for the oxidization temperatures of 1000–1200 °C (from direct weighting of specimens) and for the annealing temperatures of 1400 and 1500 °C, where the weight changes were sufficiently large to allow to be weighted accurately in crucibles. Outside these two temperature windows, the weight gains/losses were typically on the same order of magnitude as the measurement errors; thus, those data are not reported.

## Additional Information

**How to cite this article**: Gild, J. *et al*. High-Entropy Metal Diborides: A New Class of High-Entropy Materials and a New Type of Ultrahigh Temperature Ceramics. *Sci. Rep.* **6**, 37946; doi: 10.1038/srep37946 (2016).

**Publisher's note:** Springer Nature remains neutral with regard to jurisdictional claims in published maps and institutional affiliations.

## Supplementary Material

Supplementary Information

## Figures and Tables

**Table 1:** Summary of the seven metal diboride systems studied.

	Composition	Single Boride Phase?	*δ*_*a*_	*δ*_*c*_	*a* (Å)		*c* (Å)		Relative Density
					Average	XRD	Average	XRD	
HEB #1	(Hf_0.2_Zr_0.2_Ta_0.2_Nb_0.2_Ti_0.2_)B_2_	Yes	1.4%	3.9%	3.110	3.101	3.346	3.361	92.4%
HEB #2	(Hf_0.2_Zr_0.2_Ta_0.2_Mo_0.2_Ti_0.2_)B_2_	Yes	1.7%	5.2%	3.093	3.080	3.307	3.316	92.4%
HEB #3	(Hf_0.2_Zr_0.2_Mo_0.2_Nb_0.2_Ti_0.2_)B_2_	Yes	1.7%	5.2%	3.101	3.092	3.311	3.345	92.3%
HEB #4	(Hf_0.2_Mo_0.2_Ta_0.2_Nb_0.2_Ti_0.2_)B_2_	Yes	1.3%	4.0%	3.084	3.082	3.253	3.279	92.2%
HEB #5	(Mo_0.2_Zr_0.2_Ta_0.2_Nb_0.2_Ti_0.2_)B_2_	Yes	1.6%	4.6%	3.090	3.075	3.265	3.253	92.1%
HEB #6	(Hf_0.2_Zr_0.2_W_0.2_Mo_0.2_Ti_0.2_)B_2_	No	2.0%	6.2%	3.082	—	3.268	—	—
HEB #7	(Hf_0.2_Zr_0.2_Ta_0.2_Cr_0.2_Ti_0.2_)B_2_	Yes	2.3%	5.2%	3.081	3.079	3.307	3.336	92.2%

For the lattice parameters (*a* and *c*), the “average” values represent the means of five individual metal diborides while the “XRD” values represent the actual lattice parameters of the high-entropy solutions measured by XRD. See Supplementary Table S-I for additional data.
