# Supplementary material for: High-Entropy Metal Diborides: A New Class of High-Entropy Materials and a New Type of Ultrahigh Temperature Ceramics
Source: Sci Rep. 2016 Nov 29;6:37946. doi: 10.1038/srep37946 (PMC5126569; doi:10.1038/srep37946)
Supplement: Supplementary Information [file srep37946-s1.pdf]

Supplementary Information

High-Entropy Metal Diborides: A New Class of High-Entropy Materials and a New Type of Ultrahigh Temperature Ceramics

Joshua Gild[a], Yuanyao Zhang[a], Tyler Harrington[a], Sicong Jiang[a], Tao Hu[b], Matthew C. Quinn[b], William M. Mellor[b], Naixie Zhou[a], Kenneth Vecchio[a, b], Jian Luo[a, b, *]

[a]Program of Materials Science and Engineering; [b]Department of NanoEngineering

University of California, San Diego

La Jolla, CA 92093-0448, U.S.A.

Tables S-I to S-III

Figures S1 to S15

*Correspondence should be addressed to J.L. (E-mail: jluo@alum.mit.edu)

Table S-I. Summary of key parameters and measured results of the seven high-entropy metal diboride systems studied.

	Composition	Single Phase?	δ calculated from				a (Å)		c (Å)		Density (g/cm^3)		Relative Density
			a_{MB_2} (δ_a)	c_{MB_2}(δ_c)	$r_{metallic}$	$r_{covalent}$	Average	Experiment (XRD)	Average	Experiment (XRD)	Experiment	Theoretical*	
HEB #1	$(Hf_{0.2}Zr_{0.2}Ta_{0.2}Nb_{0.2}Ti_{0.2})B_2$	Yes	1.4%	3.9%	4.3%	3.5%	3.110	3.101	3.346	3.361	7.71	8.34	92.4%
HEB #2	$(Hf_{0.2}Zr_{0.2}Ta_{0.2}Mo_{0.2}Ti_{0.2})B_2$	Yes	1.7%	5.2%	5.4%	5.1%	3.093	3.080	3.307	3.316	7.87	8.52	92.4%
HEB #3	$(Hf_{0.2}Zr_{0.2}Mo_{0.2}Nb_{0.2}Ti_{0.2})B_2$	Yes	1.7%	5.2%	5.4%	5.0%	3.101	3.092	3.311	3.345	6.80	7.37	92.3%
HEB #4	$(Hf_{0.2}Mo_{0.2}Ta_{0.2}Nb_{0.2}Ti_{0.2})B_2$	Yes	1.3%	4.0%	4.4%	4.5%	3.084	3.082	3.253	3.279	7.99	8.67	92.2%
HEB #5	$(Mo_{0.2}Zr_{0.2}Ta_{0.2}Nb_{0.2}Ti_{0.2})B_2$	Yes	1.6%	4.6%	4.6%	4.5%	3.090	3.075	3.265	3.253	7.09	7.70	92.1%
HEB #6	$(Hf_{0.2}Zr_{0.2}W_{0.2}Mo_{0.2}Ti_{0.2})B_2$	No	2.0%	6.2%	6.2%	5.1%	3.082	--	3.268	--	--	--	--
HEB #7	$(Hf_{0.2}Zr_{0.2}Ta_{0.2}Cr_{0.2}Ti_{0.2})B_2$	Yes	2.3%	5.2%	7.8%	8.3%	3.081	3.079	3.307	3.336	7.36	7.98	92.2%

Note: *Theoretical densities were calculated based on the lattice parameters measured by XRD.

Table S-II. Summary of lattice parameters of the corresponding individual metal diborides (MB_2) [S1] as well as metallic (12-coordinate and at or near ambient temperatures) [S2] and covalent [S3] radii of different elements. We do not calculate δ values based on ionic radii, which depend strongly on the valence state (whereas the ionic radii for the M^{2+} in $M^{2+}(B^-)_2$ do not exist for most metals used here; additionally, the M-B bonds also have covalent characteristics and M^{2+} "cations" are highly anisotropic).

**Me**	a_{MB_2}**(Å)**	c_{MB_2} **(Å)**	$r_{metallic}$ **(Å)**	$r_{covalent}$ **(Å)**
**Zr**	3.170	3.533	1.60	1.75
**Hf**	3.139	3.473	1.59	1.75
**Nb**	3.115	3.265	1.46	1.64
**Ta**	3.088	3.241	1.46	1.70
**Mo**	3.041	3.066	1.39	1.54
**Ti**	3.038	3.220	1.47	1.60
**W**	3.020	3.050	1.39	1.62
**Cr**	2.969	3.066	1.28	1.39

References:

[S1] Y. Zhou, H. Xiang, Z. Feng, Z. Li, *J. Mater. Sci. & Tech.* 31 (2015) 285–294.

[S2] N.N. Greenwood, A. Earnshaw, Chemistry of the Elements, 2nd ed., Elsevier, 1997.

[S3] B. Cordero, V. Gómez, A.E. Platero-Prats, M. Revés, J. Echeverría, E. Cremades, F. Barragán, S. Alvarez, *Dalton Trans.* 21 (2008) 2832–2838.

Table S-III: Summary of hardness measurements of six single-phase high-entropy metal diborides, along with six individual metal diborides that were fabricated via the same HEBM and SPS route. MoB_2 is not an equilibrium bulk phase below 1500°C so that we did not have the hardness measurement on it. Thus, the "rule of mixture" averages for HEB#2-HEB#5 were calculated without MoB_2; however, MoB_2 has a lower melting temperature and theoretical hardness than five other metal diborides in question (HfB_2, ZrB_2, TaB_2, NbB_2, and TiB_2); thus, we expect the actual rule-of-mixture averages should be even lower if we could make and test a MoB_2 with the same processing conditions. Noting that the hardness can be affected by porosity and oxide inclusions, which are present in the current case due to the HEBM process that is necessary for mixing uniform high-entropy metal diborides. Thus, we choose to compare the hardness of these high-entropy diborides with individual metal diborides that were fabricated using the same method to allow a fair comparison. We expect that fully-dense and oxide-free metal diborides should have higher hardness than these measured values.

	Relative Density	Measured Hardness (GPa) Mean ± 1 St. Dev.	Rule of Mixture (Average Hardness of Individual Metal Diborides, GPa) Mean ± 1 St. Dev.
**High-Entropy Borides (HEB):**			
HEB #1: $(Hf_{0.2}Zr_{0.2}Ta_{0.2}Nb_{0.2}Ti_{0.2})B_2$	92.4%	17.5 ± 1.2	18.4 ± 1.1
MoB_2-Containing High-Entropy Diborides:			*without MoB_2
HEB #2: $(Hf_{0.2}Zr_{0.2}Ta_{0.2}Mo_{0.2}Ti_{0.2})B_2$	92.4%	19.1 ± 1.8	19.1 ± 1.3*
HEB #3: $(Hf_{0.2}Zr_{0.2}Mo_{0.2}Nb_{0.2}Ti_{0.2})B_2$	92.3%	21.9 ± 1.7	18.7 ± 0.7*
HEB #4: $(Hf_{0.2}Mo_{0.2}Ta_{0.2}Nb_{0.2}Ti_{0.2})B_2$	92.2%	22.5 ± 1.7	19.2 ± 1.3*
HEB #5: $(Mo_{0.2}Zr_{0.2}Ta_{0.2}Nb_{0.2}Ti_{0.2})B_2$	92.1%	23.7 ± 1.7	18.4 ± 1.2*
HEB #7: $(Hf_{0.2}Zr_{0.2}Ta_{0.2}Cr_{0.2}Ti_{0.2})B_2$ (overall average)	92.2%	19.9 ± 2.6	18.2 ± 1.2
HEB #7 (less dense region in the center)	>92.2%	21.0 ± 2.8	
HEB #7 (more dense region at the edge)	<92.2%	18.8 ± 1.8	
**Individual Metal Diborides (fabricated via the same HEBM and SPS route, using the same parameters#):**			
HfB_2	~80%	18.6 ± 1.3	
ZrB_2	83.7%	15.4 ± 1.1	
TaB_2	94.8%	17.5 ± 0.8	
NbB_2	96.4%	15.6 ± 0.9	
TiB_2	91.0%	25.0 ± 1.8	
CrB_2#	94.8%	14.5 ± 0.7	

#Noting an exception that CrB_2 was sintered at a lower temperature of 1800°C because its intrinsic lower melting (therefore sintering) temperature.

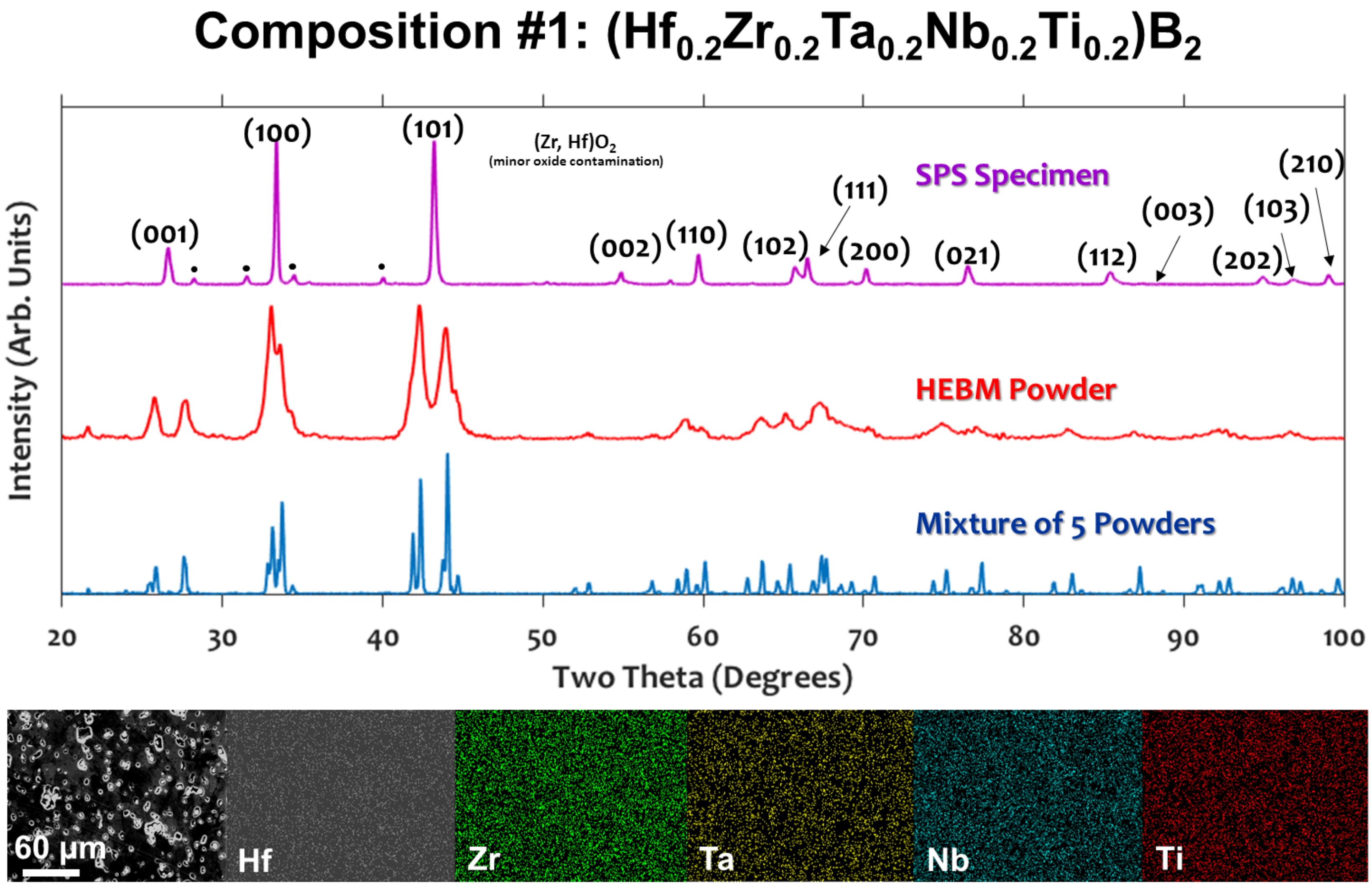

Fig. s1 Specimen #1 $(Hf_{0.2}Zr_{0.2}Ta_{0.2}Nb_{0.2}Ti_{0.2})B_2$: **(a)** XRD patterns showing phase evolution during the HEBM-SPS fabrication process. **(b)** Cross-sectional SEM image and the corresponding EDX compositional maps of the specimen after SPS.

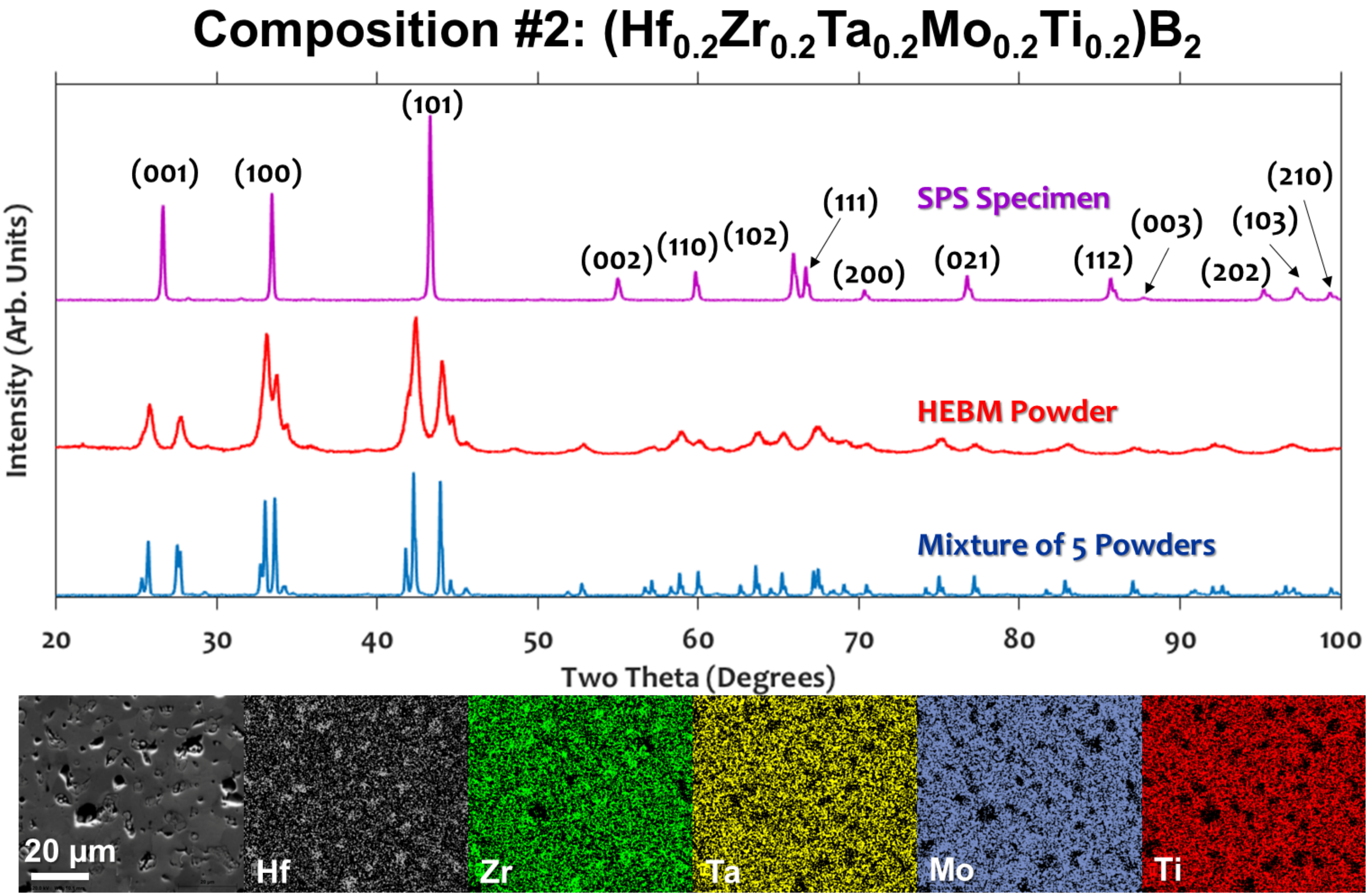

Fig. s2 Specimen #2 ($(Hf_{0.2}Zr_{0.2}Ta_{0.2}Mo_{0.2}Ti_{0.2})B_2$): **(a)** XRD patterns showing phase evolution during the HEBM-SPS fabrication process. **(b)** Cross-sectional SEM image and the corresponding EDX compositional maps of the specimen after SPS.

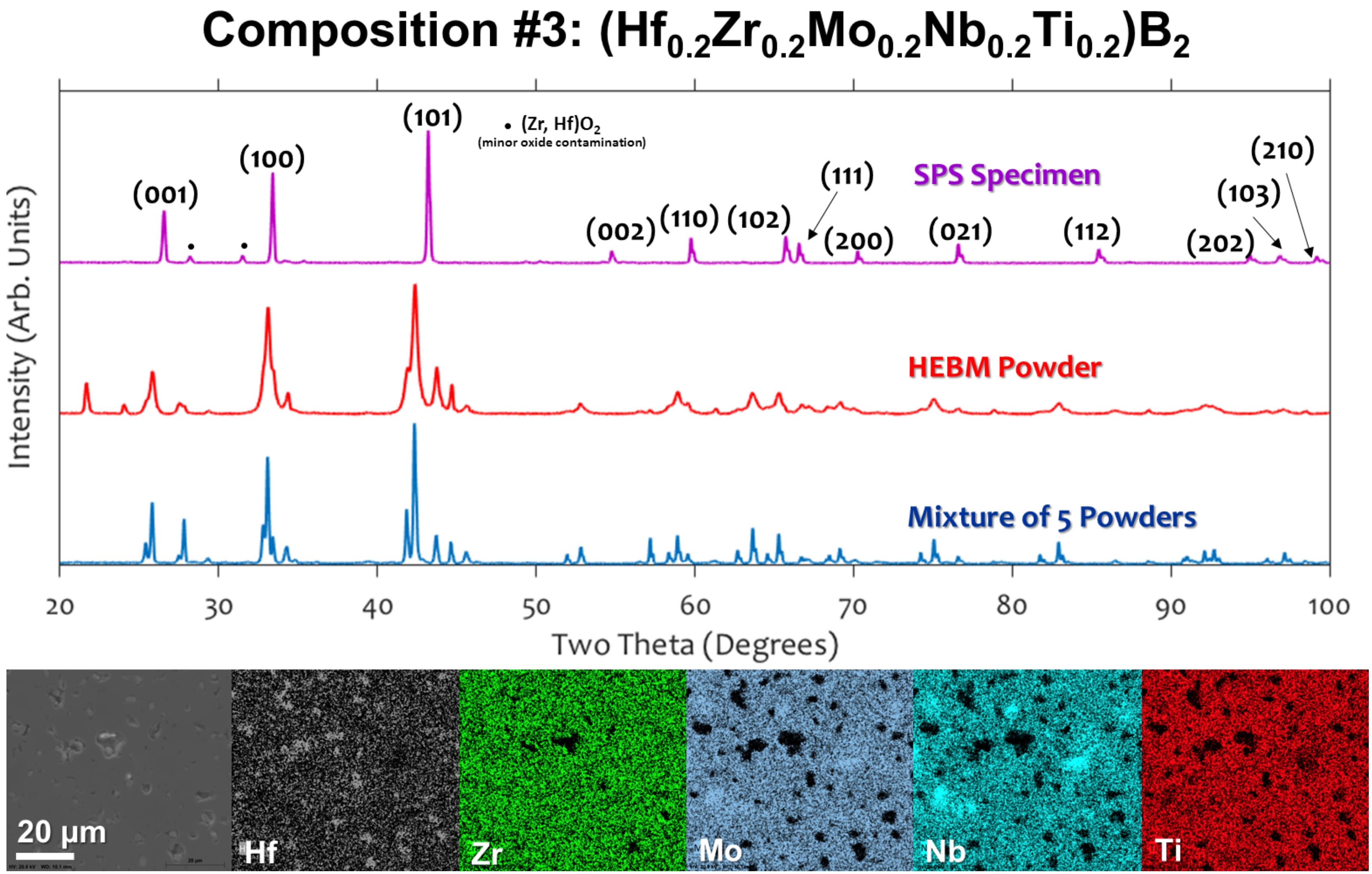

Fig. s3 Specimen #3 $(Hf_{0.2}Zr_{0.2}Mo_{0.2}Nb_{0.2}Ti_{0.2})B_2$: **(a)** XRD patterns showing phase evolution during the HEBM-SPS fabrication process. **(b)** Cross-sectional SEM image and the corresponding EDX compositional maps of the specimen after SPS.

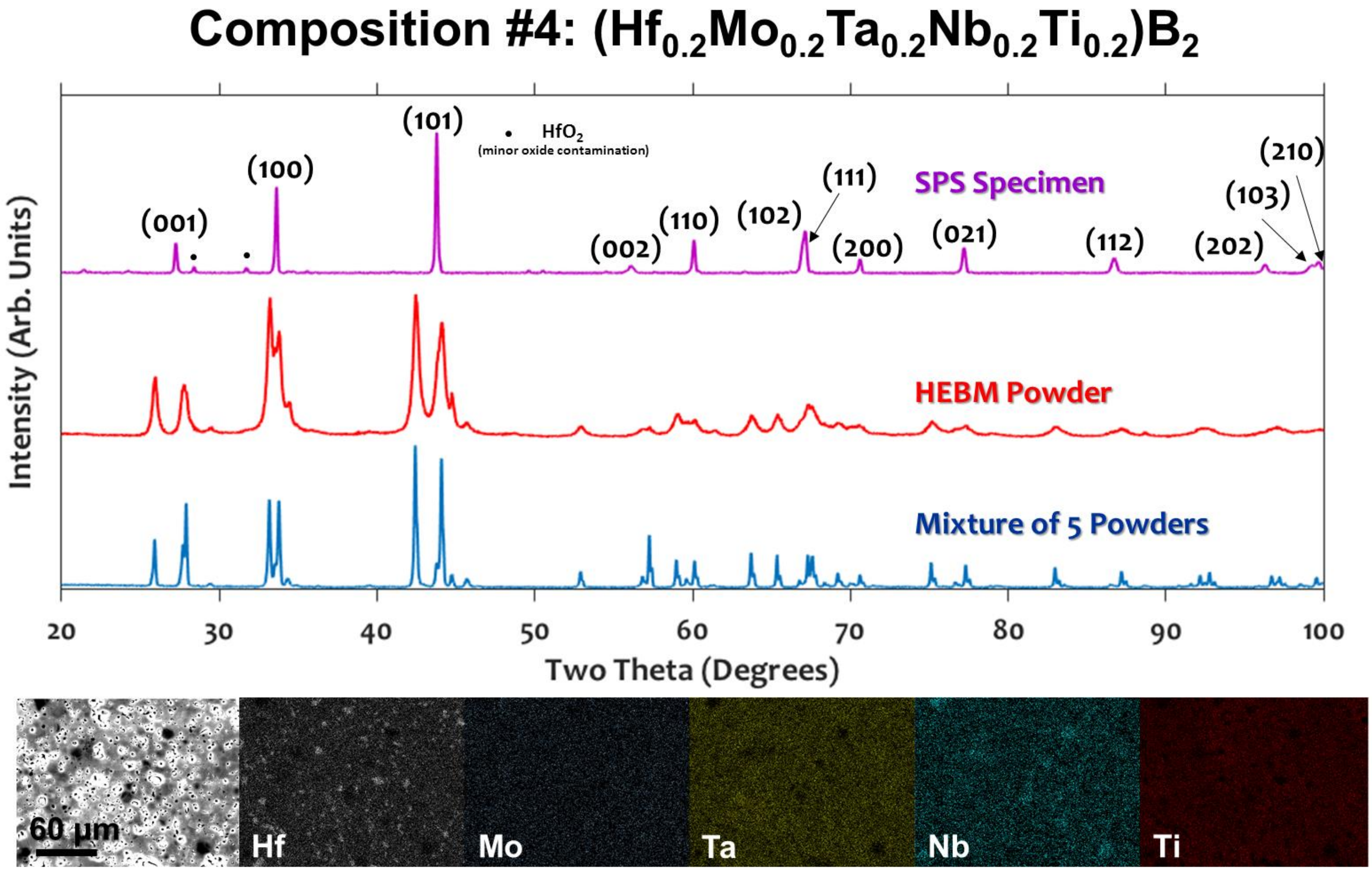

Fig. s4 Specimen #4 ($(Hf_{0.2}Mo_{0.2}Ta_{0.2}Nb_{0.2}Ti_{0.2})B_2$): **(a)** XRD patterns showing phase evolution during the HEBM-SPS fabrication process. **(b)** Cross-sectional SEM image and the corresponding EDX compositional maps of the specimen after SPS.

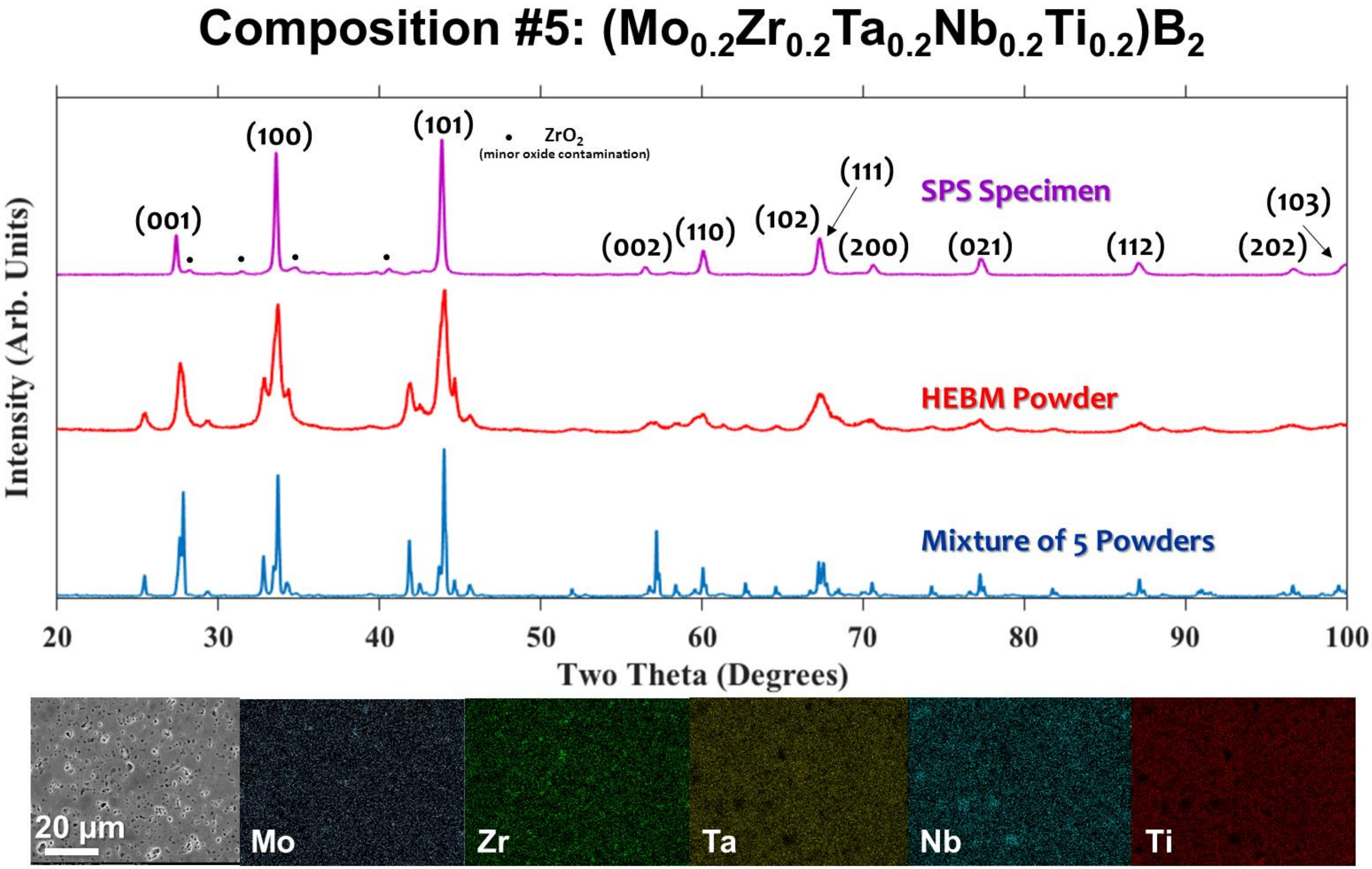

Fig. s5 Specimen #5 $(Mo_{0.2}Hf_{0.2}Ta_{0.2}Nb_{0.2}Ti_{0.2})B_2$: **(a)** XRD patterns showing phase evolution during the HEBM-SPS fabrication process. **(b)** Cross-sectional SEM image and the corresponding EDX compositional maps of the specimen after SPS.

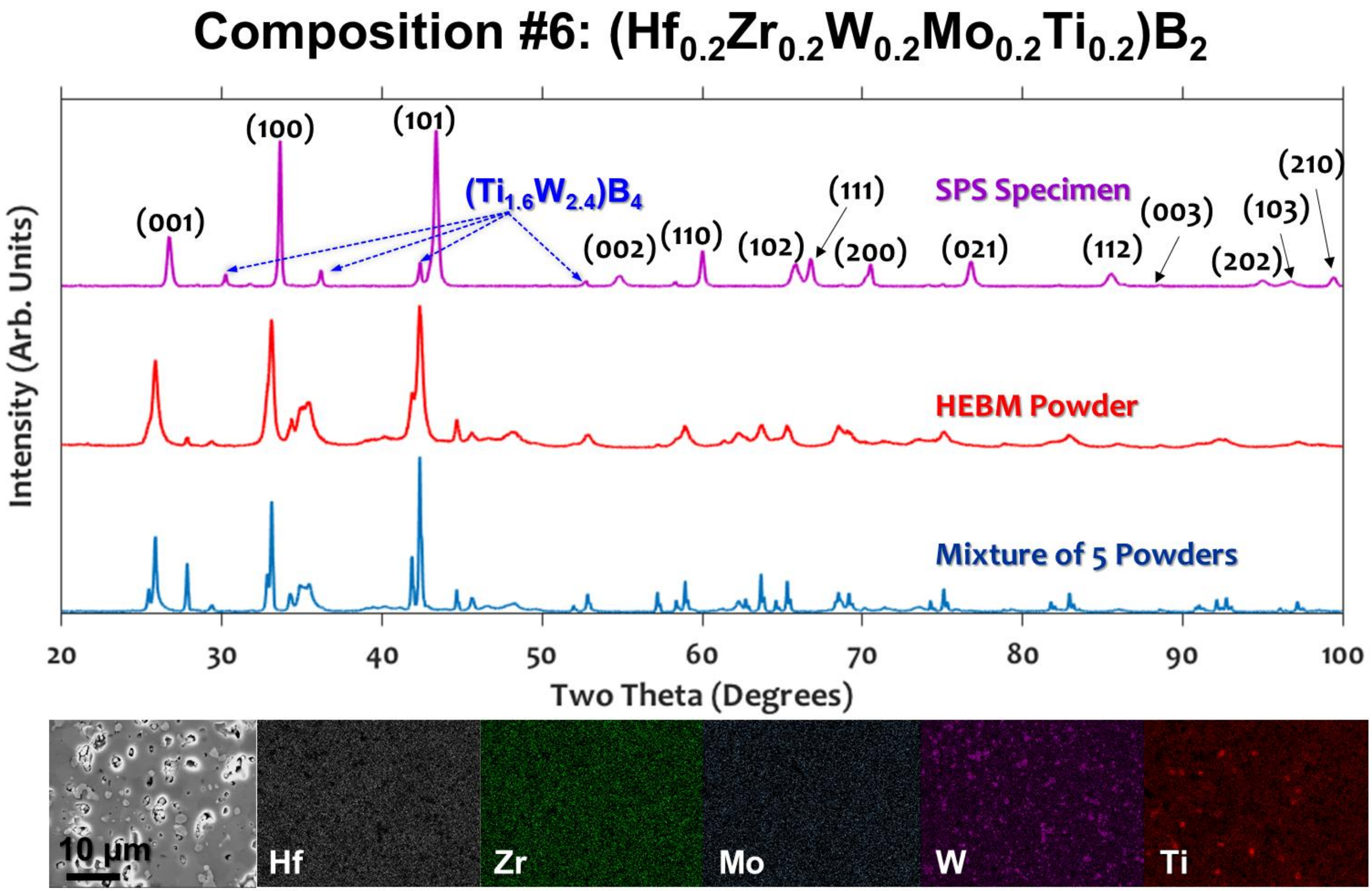

Fig. s6 Specimen #6 $(Zr_{0.2}Hf_{0.2}W_{0.2}Mo_{0.2}Ti_{0.2})B_2$: **(a)** XRD patterns showing phase evolution during the HEBM-SPS fabrication process. **(b)** Cross-sectional SEM image and the corresponding EDX compositional maps of the specimen after SPS.

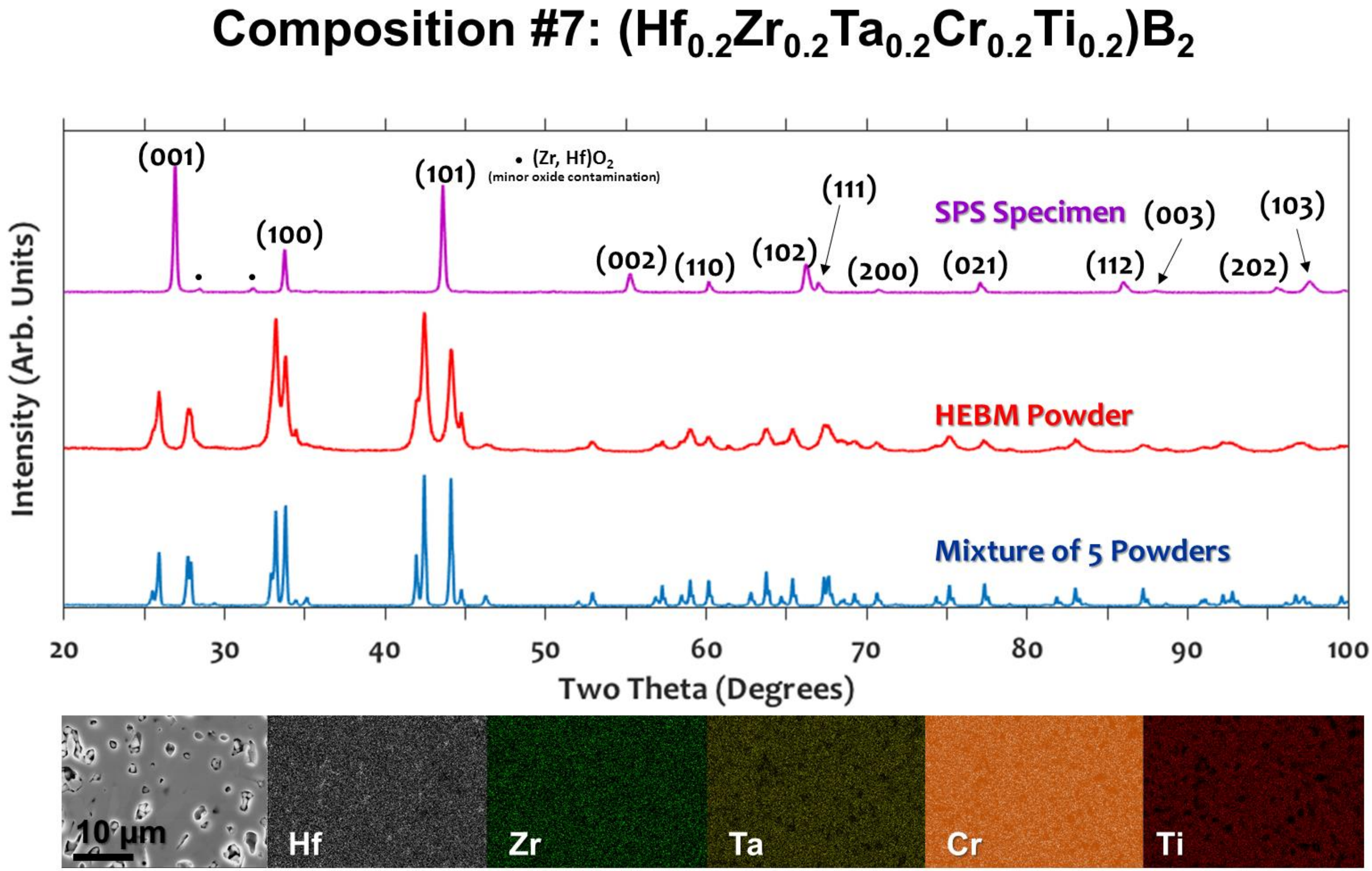

Fig. s7 Specimen #7 $(Zr_{0.2}Hf_{0.2}Ta_{0.2}Cr_{0.2}Ti_{0.2})B_2$: **(a)** XRD patterns showing phase evolution during the HEBM-SPS fabrication process. **(b)** Cross-sectional SEM image and the corresponding EDX compositional maps of the specimen after SPS.

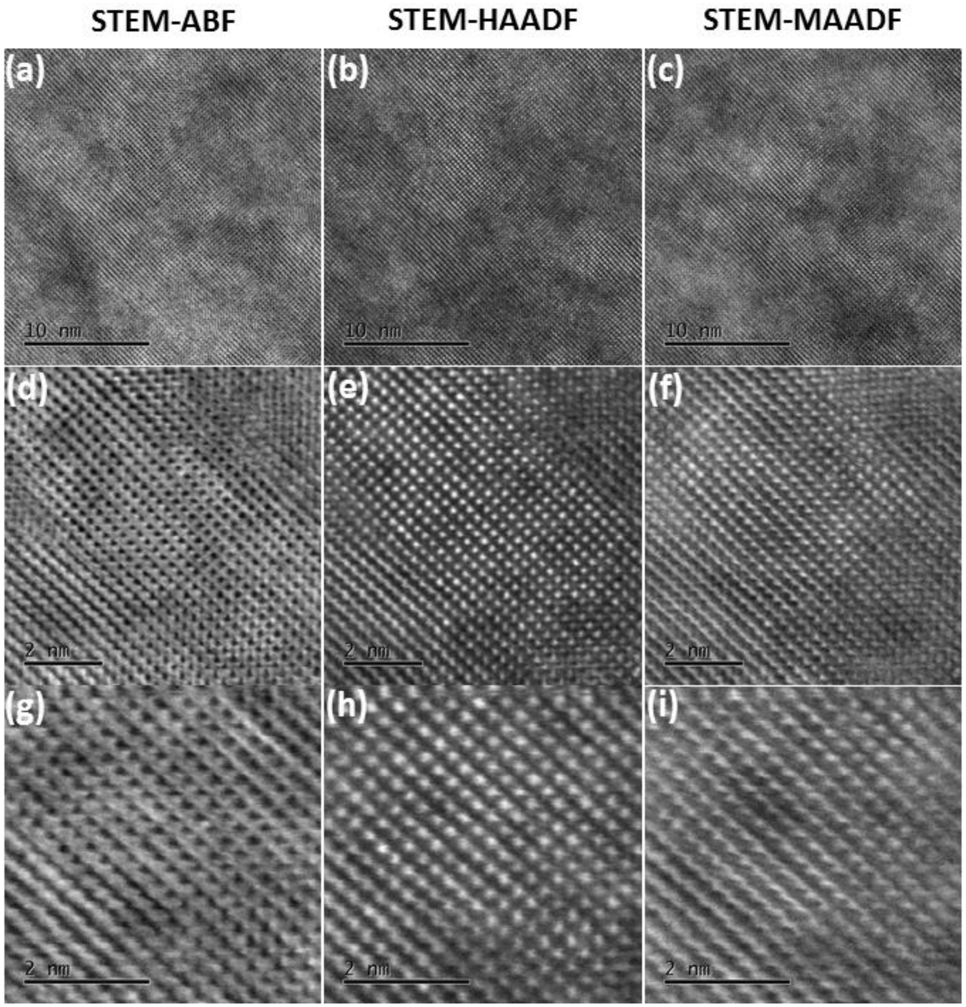

Fig. s8 STEM images at three different magnifications for Specimen #2 $(Hf_{0.2}Zr_{0.2}Ta_{0.2}Mo_{0.2}Ti_{0.2})B_2$ at a different location in the same specimen shown in Fig. 5. **(a)**, **(d)** and **(g)** are STEM-ABF images; **(b)**, **(e)** and **(h)** are STEM-HAADF images; **(c)**, **(f)** and **(i)** are STEM-MAADF images.

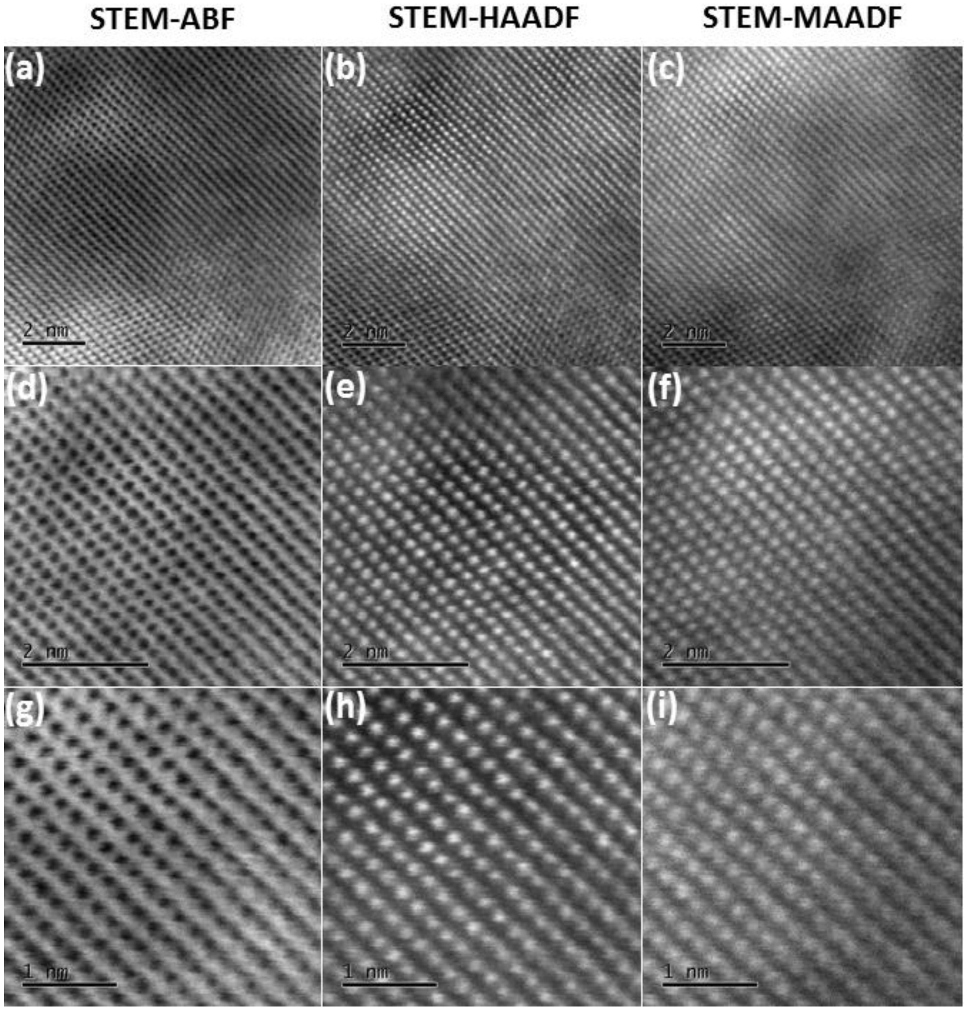

Fig. s9 STEM images at three different magnifications for Specimen #2 $(Hf_{0.2}Zr_{0.2}Ta_{0.2}Mo_{0.2}Ti_{0.2})B_2$ from a different grain in the same specimen shown in Fig. 5. **(a)**, **(d)** and **(g)** are STEM-ABF images; **(b)**, **(e)** and **(h)** are STEM-HAADF images; **(c)**, **(f)** and **(i)** are STEM-MAADF images.

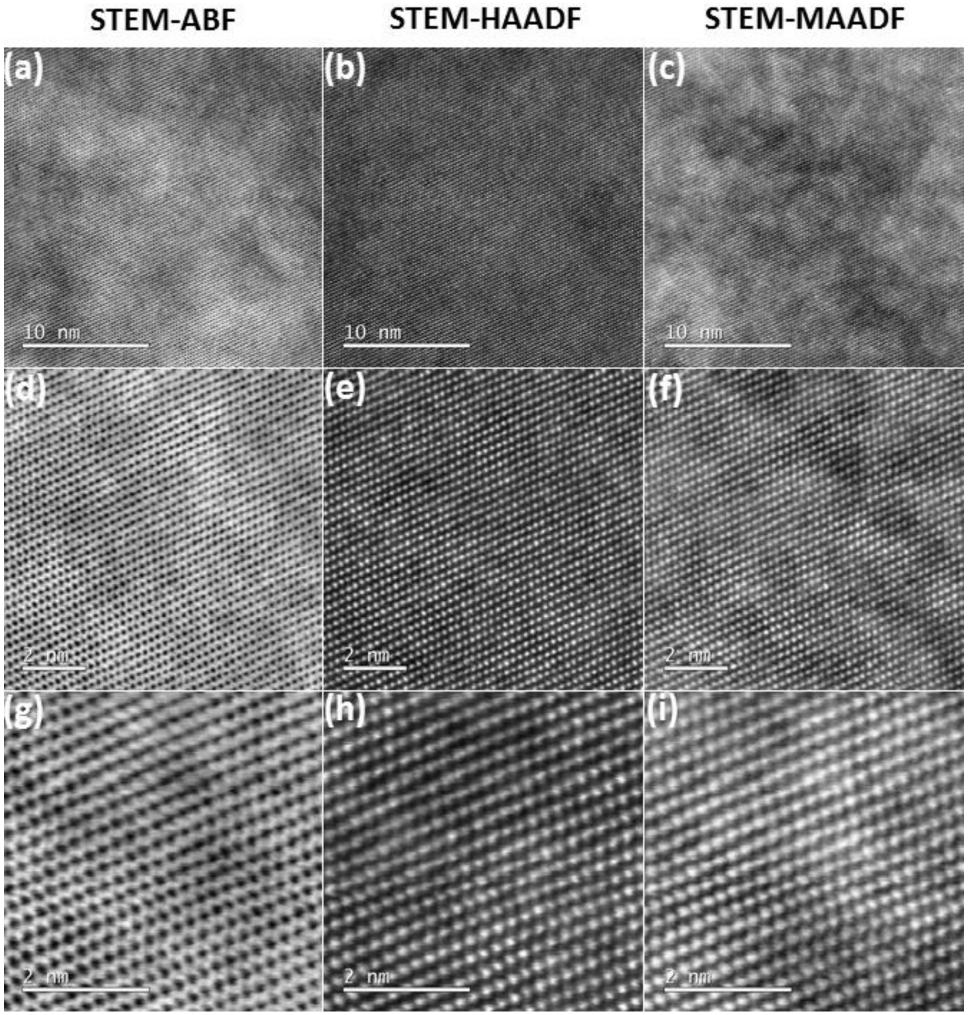

Fig. s10 STEM images at three different magnifications for Specimen #1 $(Hf_{0.2}Zr_{0.2}Ta_{0.2}Nb_{0.2}Ti_{0.2})B_2$. **(a)**, **(d)** and **(g)** are STEM-ABF images; **(b)**, **(e)** and **(h)** are STEM-HAADF images; **(c)**, **(f)** and **(i)** are STEM-MAADF images.

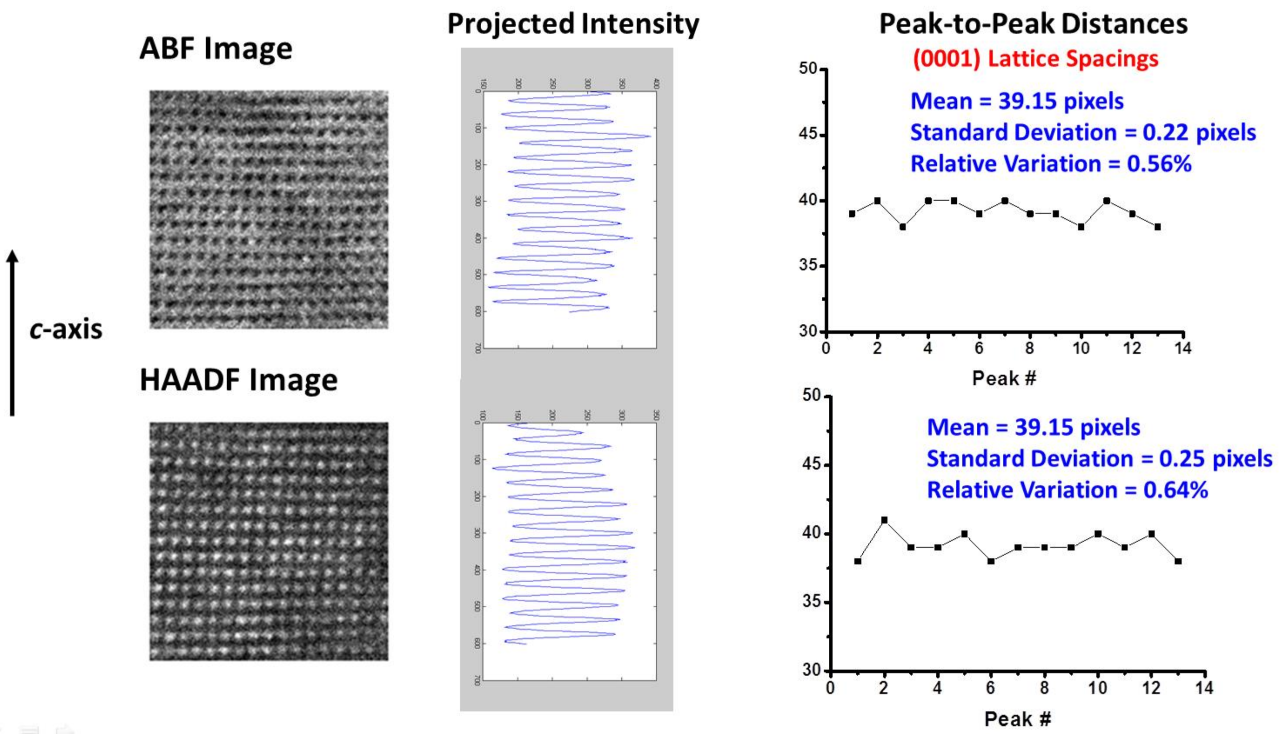

Fig. s11 Digital analysis of the STEM ADF and HAADF images from Specimen #2 $(Hf_{0.2}Zr_{0.2}Ta_{0.2}Mo_{0.2}Ti_{0.2})B_2$, showing a narrow distribution of measured basal (0001) lattice spacings that is only ~0.6% of the mean, *i.e.*, a standard variation of ~0.02 Å. This result directly confirmed the formation 2-D high-entropy metal layers between the 2-D boron nets without any significant layer-to-layer variation or segregation of different metal atoms in different 2-D metal layers.

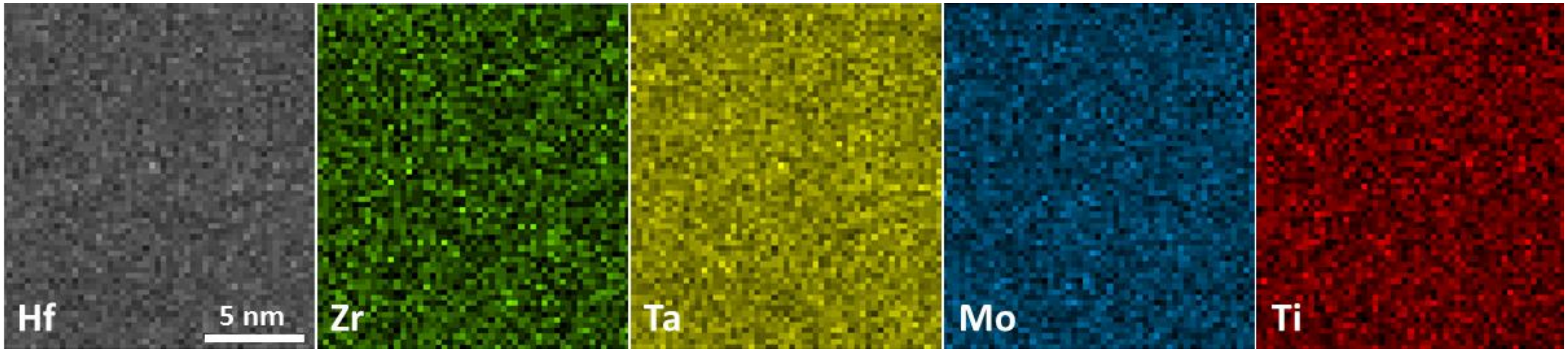

Fig. s12 Nanoscale EDX compositional maps for Specimen #2 $(Hf_{0.2}Zr_{0.2}Ta_{0.2}Mo_{0.2}Ti_{0.2})B_2$ from a different location in the same specimen shown in Fig. 6, showing the homogeneous chemical distribution at nanoscale.

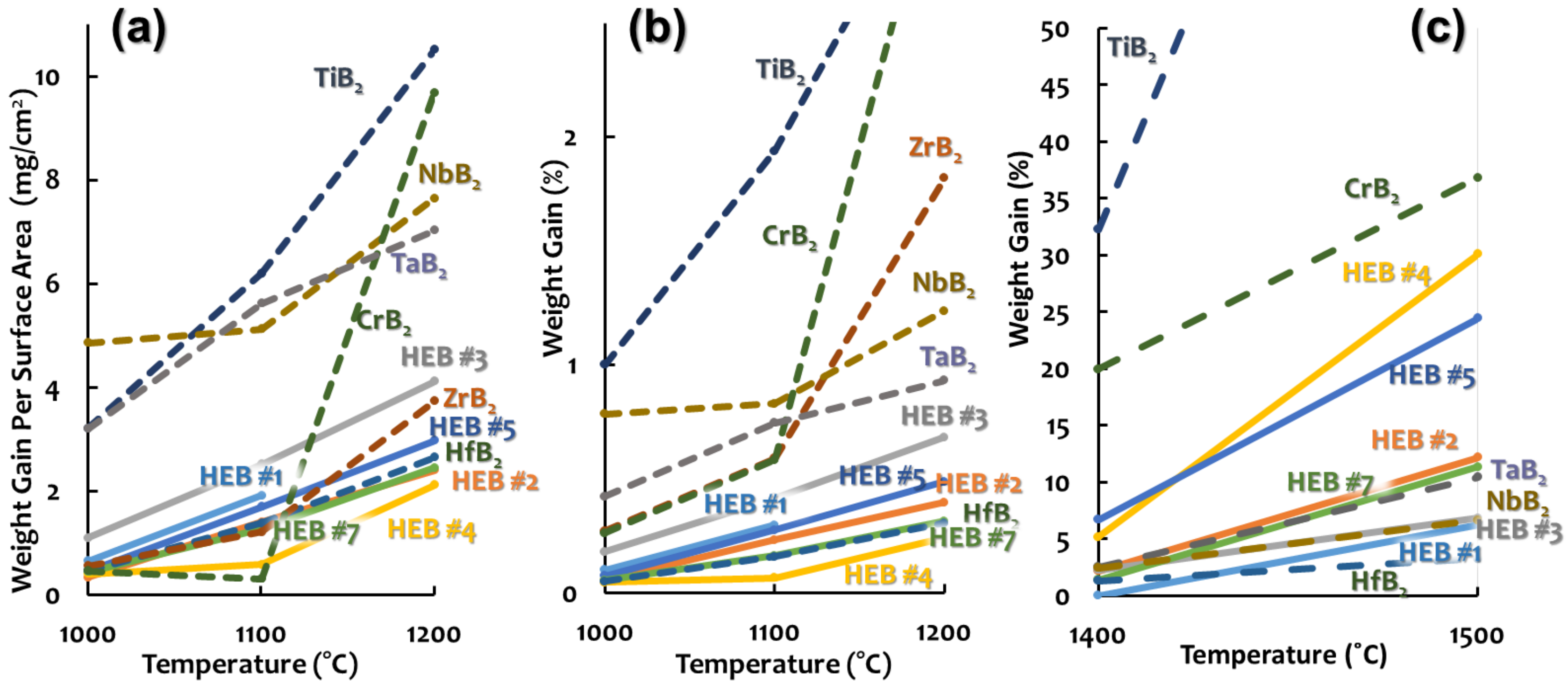

Fig. s13 (a) Weight gain per surface area and **(b)** percentage weight gain vs. oxidation temperature curves during isothermal oxidation experiments in flowing dry air at 1000°C, 1100°C, and 1200 °C (for one hour each) sequentially for six single-phase high-entropy metal diborides (*a.k.a.* HEB #1 = $(Hf_{0.2}Zr_{0.2}Ta_{0.2}Nb_{0.2}Ti_{0.2})B_2$, HEB #2 = $(Hf_{0.2}Zr_{0.2}Ta_{0.2}Mo_{0.2}Ti_{0.2})B_2$, HEB #3 = $(Hf_{0.2}Zr_{0.2}Mo_{0.2}Nb_{0.2}Ti_{0.2})B_2$, HEB #4 = $(Hf_{0.2}Mo_{0.2}Ta_{0.2}Nb_{0.2}Ti_{0.2})B_2$, HEB #5 = $(Mo_{0.2}Zr_{0.2}Ta_{0.2}Nb_{0.2}Ti_{0.2})B_2$, and HEB #7 = $(Hf_{0.2}Zr_{0.2}Ta_{0.2}Cr_{0.2}Ti_{0.2})B_2$, along with six individual metal diborides that were fabricated via the same HEBM and SPS route. **(c)** Percentage weight gain vs. oxidation temperature curves during subsequent annealing at 1400 and 1500 °C. Please see the "Methods" section for the detailed experimental procedure. A modified version of panel (b) is also presented in Fig. 8 in the main text. In all figures, solid lines represent the high-entropy metal diborides and dashed lines represent the conventional (individual) metal diborides made by the same HEBM and SPS route (as the controlled group).

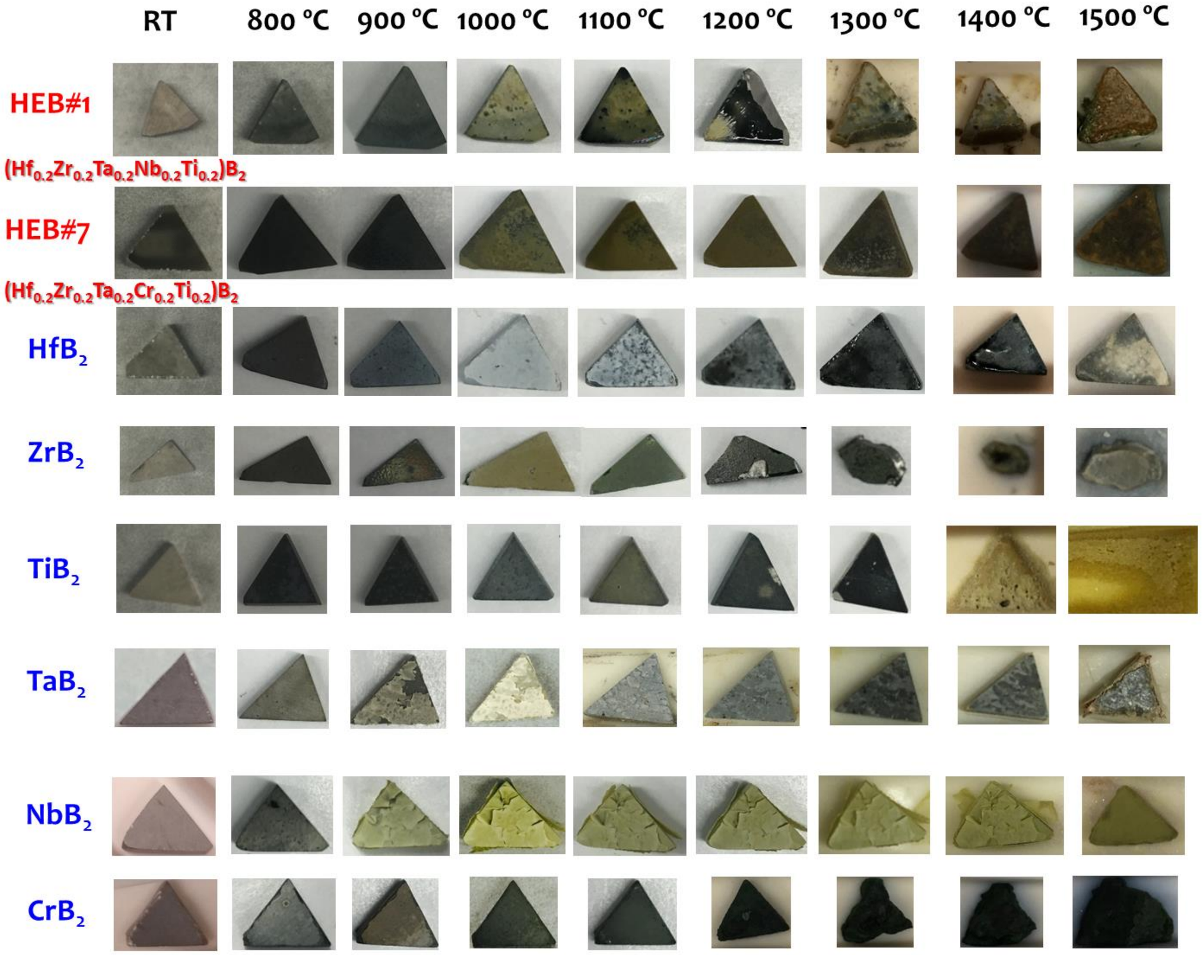

Fig. s14 Optical images showing the changes of the specimen appearance during a sequential oxidation experiments at 800-1500°C for HEB #1 $(Hf_{0.2}Zr_{0.2}Ta_{0.2}Nb_{0.2}Ti_{0.2})B_2$ and HEB #7 $(Hf_{0.2}Zr_{0.2}Ta_{0.2}Cr_{0.2}Ti_{0.2})B_2$, along with six individual metal diborides that were fabricated via the same HEBM and SPS route. Please see the "Methods" section for the detailed experimental procedure. Both HEB #1 and HEB #7 maintained their shapes up to 1500°C, while most of the six individual metal diborides (except for HfB_2) that were fabricated via the same procedure appeared to be oxidized more severely. At 1500°C, TiB_2 pulverized.

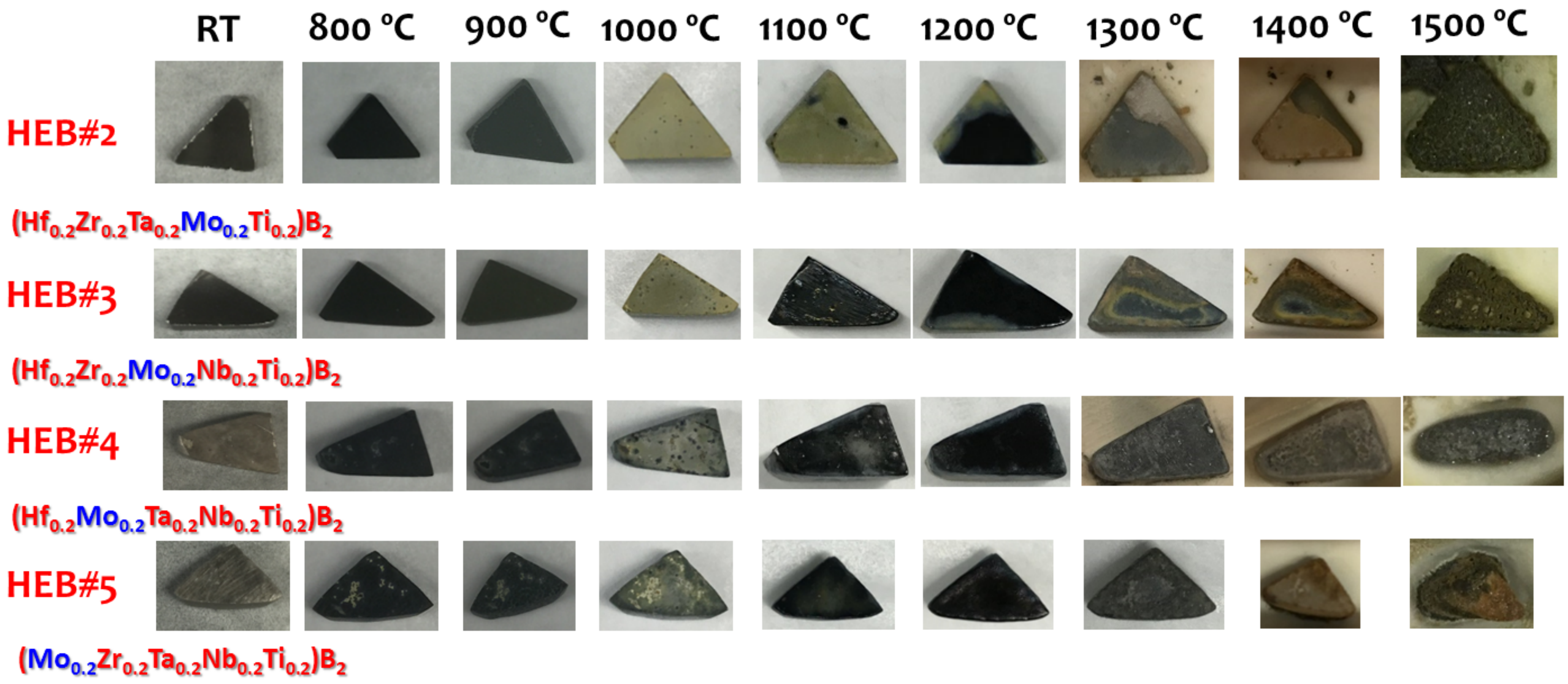

Fig. s15 Optical images showing the changes of the specimen appearance during a sequential oxidation experiments at 800-1500°C for four MoB_2-containing high-entropy diborides, which exhibited interesting, yet different, oxidation behaviors. Noting that MoO_3 is volatile in this temperature range.
